# AICAR-induced energy supply and paracrine activity of endothelial cells accelerate chronic diabetic wound healing by promoting angiogenesis and modulating macrophage reprogramming

**DOI:** 10.1016/j.mtbio.2026.103743

**Published:** 2026-10-09

**Authors:** Hengxian Su, Chang Liu, Yusheng Cheng, Wenping Cheng, Anqi Xu, Daoyuan Hu, Hongyu Shi, Mengshi Huang, Hao Yuan, Chi Huang, Yaxian Huang, Shixing Su, Chuanzhi Duan, Xin Feng

**Affiliations:** a Neurosurgery Center, Department of Cerebrovascular Surgery, National Key Clinical Specialty, Engineering Research Center for Diagnostic and Therapeutic Technology and Devices for Cerebrovascular Diseases under the Ministry of Education, Guangdong Provincial Key Laboratory for Brain Function Repair and Regeneration, Zhujiang Hospital Institute for Brain Science and Intelligence, Zhujiang Hospital, Southern Medical University, Guangzhou, 510282, China; b Department of Orthopedic Surgery, The Lingnan Hospital of Sun Yat-Sen University, Guangzhou, 510630, China; c Department of General Surgery, The Second Clinical Medical College, Lanzhou University, Lanzhou, 730030, China; d Department of Urology, Biomedical Innovation Center, The Sixth Affiliated Hospital, Sun Yat-Sen University, Guangzhou, 510655, China

**Keywords:** Chronic diabetic wound, AICAR, High-energy state, Paracrine, Exosomes, Macrophage reprogramming

## Abstract

Chronic diabetic wound healing is severely hampered by endothelial cell (EC) dysfunction, defective angiogenesis, and a persistent inflammatory microenvironment, yet the crosstalk between EC energy metabolism and macrophage polarization in this process remains poorly understood. Here, we identified an AMPK-dependent metabolic pathway in ECs that orchestrated both angiogenic restoration and macrophage immune reprogramming for diabetic wound repair, and translated this mechanism into a functional three-dimensional (3D) scaffold (MEWs + GelMA@AI) fabricated by melt electrospinning writing technology. Acadesine (AICAR) efficiently induced a high-energy state in ECs by activating AMPK, preserving mitochondrial ultrastructure, enhancing respiratory-chain complex activity, and increasing VEGF expression and angiogenic potential. Notably, we discovered that AI-EXO, secreted by these metabolically activated ECs, delivered GAS6 to macrophages as a key paracrine factor, thereby triggering activation of the MER/AKT pathway and driving M1-to-M2 polarization. To our knowledge, this is the first report identifying exosomal GAS6 as a mediator of EC-macrophage crosstalk in diabetic wound healing. The MEWs + GelMA@AI scaffold, characterized by a porous structure and sustained AICAR release, exhibited excellent cytocompatibility and proangiogenic activity *in vitro*. *In vivo*, the scaffold significantly accelerated diabetic wound closure by promoting neovascularization, reducing inflammatory infiltration, and increasing the M2/M1 macrophage ratio, without detectable systemic toxicity in major organs. These findings establish a critical link between EC energy metabolism and immune microenvironment remodeling, providing a potentially translatable metabolic-engineering strategy for chronic diabetic wounds. This study opens new avenues for regenerative therapies targeting metabolic-immune interactions.

## Graphical abstract

**Figure:**
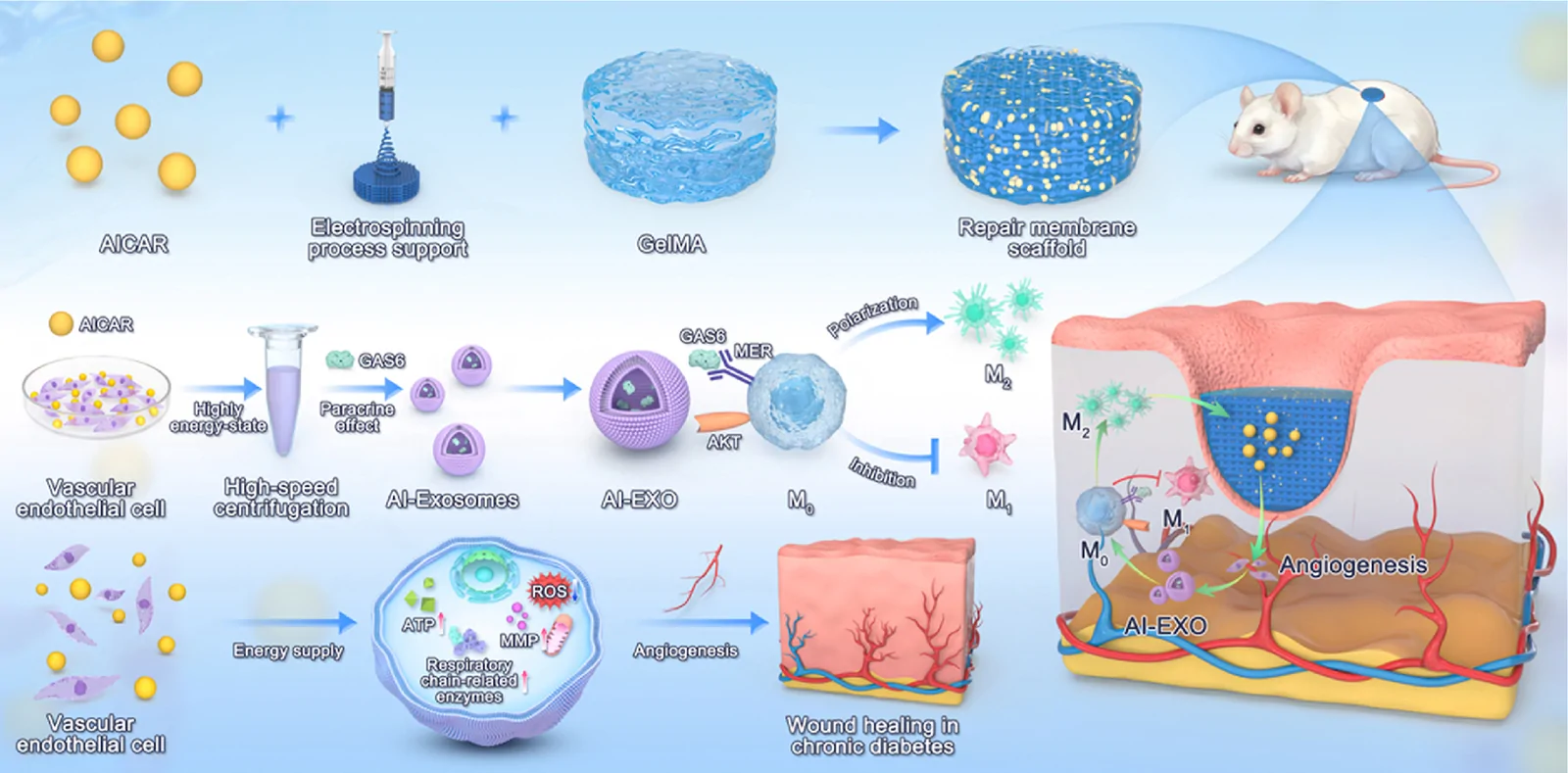

## 1 Introduction

Diabetes mellitus, a chronic metabolic disorder characterized by endocrine dysfunction and persistent hyperglycemia, is among the most prevalent and rapidly growing global health challenges, with 693 million adults projected to be affected by 2045 [1], [2]. The lifetime risk of developing a diabetic wound is approximately 25% among patients with diabetes, representing a substantial and persistent challenge in tissue repair [3]. The poor healing of chronic diabetic wounds is primarily attributable to aberrant angiogenesis and a dysregulated immune microenvironment and frequently leads to limb amputation and substantial morbidity [4], [5]. Despite advances in wound care, current therapies often fail to address the underlying pathophysiological complexity [6]. Accordingly, dual-targeted approaches that simultaneously normalize the immune microenvironment and foster vascularization have thus become a major focus of therapeutic development.

Macrophages, as pivotal components of the immune system, play an integral role in maintaining tissue homeostasis, particularly during the processes of tissue repair and regeneration [7]. These innate immune cells exhibit remarkable plasticity and adapt their functional states in response to various environmental cues. This plasticity enables macrophages to transition between pro-inflammatory (M1) and anti-inflammatory (M2) phenotypes, each of which contributes distinctly to the healing process [8], [9], [10]. In diabetes, the transition from M1 to M2 is impaired, perpetuating a destructive inflammatory milieu [11]. The reprogramming of macrophages has emerged as a critical therapeutic target for diabetic wound healing. For example, Shu et al. developed a netrin-1-releasing GelMA hydrogel and demonstrated that netrin-1 accelerates diabetic wound healing by inducing M1-to-M2 macrophage repolarization [12]. Wang et al. developed a silk fibroin/gelatin (SG/M) hydrogel containing Mdivi-1, which significantly accelerated diabetic wound healing by promoting macrophage polarization toward the M2 phenotype [13]. Although these findings support M1-to-M2 repolarization as a viable strategy for correcting immune dysregulation and improving diabetic wound healing, single-target interventions remain insufficient to address the complex pathophysiology of diabetes.

The balance between M1 and M2 macrophage populations directly influences the outcomes of regeneration, underscoring the importance of understanding the mechanisms that drive macrophage reprogramming. Recent advances in cellular and molecular biology have elucidated the signaling pathways and transcriptional networks involved in this transition, revealing potential therapeutic targets [10]. Strategic modulation of macrophage phenotypes may optimize the regenerative microenvironment and improve outcomes in chronic wounds, heart disease, and neurodegenerative disorders [14], [15], [16], [17]. Understanding the interactions between macrophage reprogramming and other cellular components of the regenerative milieu is therefore essential for developing integrated approaches to diabetic wound repair. In this context, ECs are indispensable for neovascularization and wound repair and represent an important therapeutic target. Investigating EC-macrophage crosstalk may therefore provide a particularly promising strategy for improving chronic diabetic wound healing.

Our previous study demonstrated that functionalizing artificial small-diameter blood vessels with AICAR could promote angiogenesis by activating AMPK and increasing EC energy levels. Notably, we observed greater accumulation of M2 macrophages around the grafts, which contributed to improved graft survival [18]. However, the underlying mechanisms remained unclear, and the application of this strategy to diabetic wound healing had not been reported. Physiologically, an enhanced metabolic state facilitates anabolic biosynthesis and mitosis, thereby accelerating regeneration [19], [20]. Liu et al. engineered bioenergetic-active exosomes (Suc-EXO) to promote cartilage regeneration and maintain homeostasis [21]. These exosomes exhibited a 5.42-fold rise in ATP content and modulated cellular energy metabolism by promoting the tricarboxylic acid cycle, thereby facilitating the chondrogenic differentiation of bone marrow mesenchymal stem cells [21]. Moreover, Zhang et al. developed a versatile class of bioinks and achieved rapid 3D printing of bioenergetic-active scaffolds (BASs) designed for orthopedic applications. These scaffolds released bioenergetic units that enhanced cellular metabolic states and promoted cell differentiation and tissue regeneration [22]. Nevertheless, previous studies have not elucidated the relationship between cellular energy metabolism and vascularization during tissue regeneration, and the mechanisms linking cellular energy metabolism to diabetic wound healing remain unclear.

Cellular energy metabolism supplies the energy required for cell proliferation, migration, and differentiation, all of which are essential for tissue angiogenesis [23]. Metabolic reprogramming via AMPK activation represents an emerging paradigm in regenerative medicine [24]. AICAR, an AMPK agonist, can induce a high-energy state characterized by enhanced mitochondrial oxidative phosphorylation (OXPHOS) and ATP production [25]. Augmenting the energy supply of ECs may therefore be beneficial for treating chronic diabetic wounds.

Exosomes, nano-sized extracellular vesicles that carry bioactive cargo, have emerged as critical mediators of intercellular communication in tissue regeneration [26], [27], [28], [29] and show particular promise for modulating immune responses [30], [31], [32]. Building on this foundation, we hypothesized that exosomes derived from high-energy-state ECs (AI-EXO) carry distinct protein cargo capable of reshaping macrophage function through metabolic-immune crosstalk. In this study, we performed proteomic profiling of exosomes derived from AICAR-induced ECs to identify differentially expressed proteins and elucidate the mechanism of endothelial-macrophage crosstalk in diabetic wound healing. We subsequently developed a MEWs + GelMA@AI patch and evaluated its efficacy in a chronic diabetic wound model in rats, in which it markedly promoted healing. Our findings provide new insights into therapeutic strategies addressing the multifactorial pathology of diabetic wounds.

## 2 Materials and methods

### 2.1 Cell culture

Commercially available Human umbilical vein endothelial cells (HUVECs) and RAW264.7 macrophages were purchased from Sciencell (San Diego, CA, USA). Cells were cultured in Minimum Essential Medium (MEM, Gibco) supplemented with 10% fetal bovine serum (FBS, Exosome-depleted serum, Gibco) at 37 °C in a humidified atmosphere containing 5% CO_2_. Consistent with our previous dose-response characterization, 1 mM AICAR was identified as the effective concentration and was used in subsequent experiments [18]. To establish inflammatory conditions, HUVECs or RAW264.7 cells (passages 3-8) were exposed to 10 μg/mL LPS for 24 h before subsequent assays. The effects of AI-EXO at concentrations ranging from 1 × 10^7^ to 5 × 10^9^ particles/mL on RAW264.7 cell viability were evaluated. A concentration of 5 × 10^8^ particles/mL was selected (Fig. S1) and used in subsequent experiments.

### 2.2 Fabrication of melt electrospinning writing scaffolds (MEWs)

MEWs scaffolds were fabricated using a 3D extrusion-based melt electrospinning system (Medprin, Guangzhou, China). Polycaprolactone (PCL, MW: 80 kDa) was melted at 150 °C in a melt barrel and extruded through a 23-G nozzle using a high-voltage device (Medprin Biotech GmbH, Germany). The optimized operational parameters included: spinning voltage, 2.2-3 kV; receiving distance, 2-3 mm; receiving-plate speed, 5 mm/s; and temperature, 5 °C. The layer height was approximately 40 μm, the printing speed was 20 mm/s, and the air-pump pressure was maintained at 15-25 kPa. The scaffolds were washed three times with deionized water, yielding final constructs 1.5 cm in diameter and 500 μm in height.

### 2.3 Preparation of MEWs + GelMA@AI

Lyophilized GelMA (EFL-GM-60, Suzhou, China) and photoinitiator were dissolved in PBS to prepare a 10% (w/v) GelMA solution containing 0.05% photoinitiator. AICAR was added at a final concentration of 1 mM to prepare the GelMA@AI solution. Porous MEWs scaffolds were loaded with 120 μL GelMA@AI solution, followed by photo-crosslinking under UV light at a distance of 10 cm for 2 min to form stable MEWs + GelMA@AI constructs. MEWs + GelMA control scaffolds were fabricated using the same protocol without AICAR.

### 2.4 Characterization of MEWs + GelMA@AI

#### 2.4.1 Scanning electron microscopy (SEM)

MEWs and MEWs + GelMA@AI scaffolds were dehydrated through a graded ethanol series (70%, 80%, 90%, 95%, and 100%; 10 min each), lyophilized (Telstar, Spain), and sputter-coated with gold before SEM analysis (Carl Zeiss, Germany). The internal scaffold structure was examined after careful sectioning with a sharp scalpel.

#### 2.4.2 AICAR release profile

GelMA@AI or MEWs + GelMA@AI constructs were immersed in 2 mL PBS at 37 °C. At predetermined time points (days 3, 6, 9, 12, 15, 18, and 21), 100 μL of supernatant was collected for AICAR concentration measurement using established methods [18].

#### 2.4.3 Fourier transform infrared spectroscopy (FTIR)

Samples were dehydrated and lyophilized before Fourier transform infrared spectroscopy (FTIR; Tianjin, China). Spectra were acquired in transmittance mode over the range of 400-3600 cm^−1^, and characteristic peaks of AICAR, GelMA, and PCL were identified and marked.

#### 2.4.4 Compressive modulus characterization

The compressive mechanical behaviors of pure GelMA, MEWs, MEWs + GelMA, and MEWs + GelMA@AI were assessed via quasi-static uniaxial compression using a universal mechanical tester (MTS E43.104, Shenzhen, China). Before testing, all samples were equilibrated fully in PBS at 37 °C for 24 h to simulate physiological wet conditions. A preload of 0.05 N was applied to ensure complete surface contact, followed by compression at a constant rate of 1 mm/min up to a maximum strain of 15%. The compressive modulus was determined from the linear elastic region of the stress-strain curve at 2-10% strain. Six replicates per group were analyzed.

#### 2.4.5 Equilibrium swelling ratio

The equilibrium swelling ratio (ESR) of pure GelMA, MEWs, MEWs + GelMA, and MEWs + GelMA@AI was measured in PBS at 37 °C. All samples were first vacuum-dried to a constant weight to record the dry mass (W_d_). Samples were then immersed in PBS and incubated until the swelling equilibrium was reached. Excess surface water was gently removed with filter paper before the swollen samples were weighed (W_s_). The equilibrium swelling ratio was calculated as ESR=W_s_/W_d_. Six replicates were analyzed for each group.

### *2.5* *In vitro* cytocompatibility assessment of the MEWs + GelMA@AI

HUVEC viability was assessed using a fluorescent live/dead assay kit (Nanjing, China). Inflammatory HUVECs (2 × 10^5^ cells/well) were incubated with MEWs + GelMA (control) or MEWs + GelMA@AI (AICAR group) for 24 h in 24-well plates. Compound C, an AMPK inhibitor, was used to block AICAR activity in inhibitor groups. Cells were stained according to the manufacturer's instructions and imaged by laser-scanning confocal microscopy (LSM 880, Zeiss, Jena, Germany) to visualize live (green) and dead (red) cells. For cell-infiltration analysis, FITC-phalloidin staining was performed, nuclei were counterstained with DAPI, and images were acquired by confocal microscopy.

### 2.6 Measurement of mitochondrial function

HUVECs were incubated with scaffolds for 24 h, then harvested after gentle PBS washing. Trypsin (0.25% w/v) was used for cell detachment, with GelMA lysis solution (EFL-GM-LS-001, Suzhou, China) for complete dissociation from GelMA-containing scaffolds. Transmission electron microscopy (TEM) was used to examine mitochondrial ultrastructure after fixation, dehydration, embedding, ultrathin sectioning, and uranium staining. Mitochondrial function was assessed using: MitoTracker Green staining for mitochondrial mass, JC-1 staining and flow cytometry for mitochondrial membrane potential (MMP), ATP detection assays for energy production, and MitoSOX™ Red staining (Beyotime, Shanghai, China) for mitochondrial superoxide levels. All assays were performed according to the manufacturers' instructions.

### 2.7 Determination of NAD^+^/NADH and lactate/pyruvate ratios

#### *2.7.1* *In vitro* assays in HUVECs

HUVECs were pretreated with 10 μg/mL LPS for 24 h and then incubated with 1 mM AICAR, with or without 10 μM Compound C, for another 24 h. Cells were lysed in ice-cold lysis buffer, and the supernatants were collected after centrifugation at 12,000 rpm for 10 min at 4 °C. The NAD^+^/NADH ratio was measured using a colorimetric NAD^+^/NADH Assay Kit (MedChemExpress, NJ, USA). Lactate and pyruvate concentrations were quantified using commercial colorimetric assay kits (Beyotime, Shanghai, China) according to the manufacturer's protocols. The NAD^+^/NADH ratio and lactate/pyruvate ratio were then calculated.

#### *2.7.2* *In vivo* assays in wound tissues

Full-thickness wound tissues were harvested on post-wounding days 3, 7, and 14, immediately snap-frozen in liquid nitrogen, and stored at −80 °C. Approximately 20 mg of frozen tissue was homogenized in pre-cooled lysis buffer on ice. After centrifugation, the supernatants were collected and analyzed using the same assay kits used for the *in vitro* experiments.

### *2.8* *In vitro* study of angiogenesis

#### 2.8.1 Western blot (WB)

HUVECs from different scaffold groups were lysed in cell lysis buffer containing 1 mM phenylmethanesulfonyl fluoride (Beyotime, Shanghai, China). Protein concentrations were determined using the BCA assay. Forty micrograms of protein were separated by 12% SDS-PAGE at 100 V for 1.5 h and transferred to 0.45-μm nitrocellulose membranes. Membranes were blocked with 5% non-fat milk for 1 h at room temperature (RT), then incubated with primary antibodies against VEGF, AMPK, and p-AMPK (Thr 172) (1:1000; Affinity Biosciences, Jiangsu, China) for 24 h at 4 °C. Goat anti-Rabbit IgG (H + L) (1:10,000, Affinity Biosciences) was used as a secondary antibody. Protein bands were visualized using an Affinity ECL kit and quantified with ImageJ software (National Institutes of Health, USA).

#### 2.8.2 Immunofluorescence staining of VEGF

HUVECs were fixed with 4% paraformaldehyde for 15 min at room temperature, permeabilized with 0.2% Triton X-100 for 10 min, and blocked with 5% normal goat serum for 1 h at room temperature. Cells were incubated overnight at 4 °C with a rabbit anti-VEGF antibody (1:500; Affinity Biosciences), followed by an Alexa Fluor 488-conjugated secondary antibody (1:500; 1 h at room temperature) and DAPI nuclear counterstaining. Confocal images were acquired using an LSM 880 microscope (Zeiss) and analyzed with ImageJ.

#### 2.8.3 Scratch assay

HUVECs were cultured to confluence in the lower chambers of 24-well Transwell plates. After 24 h incubation, linear scratches were created using a 200-μL pipette tip, with cell debris removed by PBS washing. The medium was replaced with serum-free MEM, and the scaffolds were placed in the upper chambers. After an additional 24 h, the scratches were photographed, and cell migration was quantified using ImageJ according to the following formula: scratch healing (%) = (S1 - S2)/S1 × 100%, where S1 and S2 represent scratch areas before and after incubation, respectively.

#### 2.8.4 Chick chorioallantoic membrane (CAM) assay

Fertilized eggs were maintained under standard conditions. A small opening was created at the blunt end of the eggshell without damaging the underlying chorioallantoic membrane (CAM) and was then sealed with tape to prevent contamination. Extracts from the experimental MEWs scaffold groups were applied to the CAM for 3 days, after which angiogenic changes were observed and quantified.

#### 2.8.5 Tube formation assay

Matrigel (100 μL) was thawed overnight at 4 °C, added to 24-well plates, and allowed to solidify at 37 °C for 60 min. HUVECs (2 × 10^5^ cells) were seeded onto the Matrigel, and the scaffolds were placed in the upper chambers. After 12 h of incubation at 37 °C in 5% CO_2_, tubular structures were visualized using an optical microscope. Angiogenic parameters, including mesh number, master segments, master junctions, and total segment length, were quantified using the ImageJ Angiogenesis Analyzer.

### 2.9 Transient transfection

To obtain AI-EXO^@Si−GAS6^, siRNA targeting human GAS6 (Si-GAS6) and a negative-control siRNA (Si-NC) were commercially obtained from GenePharma (Shanghai, China). HUVECs were transiently transfected with siRNA using Lipofectamine 3000 (Invitrogen, USA). The Si-GAS6 sequences were as follows:

SiRNA-1: 5′-GCCUCCAGAUCUGCCACAATT-3′,

SiRNA-2: 5′-CCUGACCGUGGGAGGUAUUTT-3′,

SiRNA-3: 5′-GCAUACGGCCUUGGCCCUATT-3'.

SiRNA-NC: 5′-UUCUCCGAACGUGUCACGUTT-3'.

GAS6 knockdown was confirmed by western blotting 48 h after transfection.

### 2.10 Isolation, characterization, and bioinformatic analysis of AI-EXO and N-EXO

HUVECs cultured under inflammatory conditions were maintained in MEM supplemented with 10% FBS under standard conditions (37 °C and 5% CO_2_), and the medium was replaced every 48 h. At 80% confluence, the cells were cultured for 48 h in serum-free medium containing 1 mM AICAR. Parallel control cultures received the same serum-free medium without AICAR. The supernatants were collected and initially centrifuged at 300 × g for 10 min at 4 °C to remove dead cells. Exosomes were then isolated by sequential centrifugation as follows: 2000 × g for 30 min to remove cell debris and apoptotic bodies; 10,000 × g for 45 min to remove large vesicles; and 120,000 × g for 70 min at 4 °C. The pellet was resuspended in PBS and purified by a second ultracentrifugation step under the same conditions. Exosomes derived from AICAR-preconditioned HUVECs (AI-EXO) or parallel control cells (N-EXO) were resuspended in 200 μL of PBS for subsequent experiments.

The morphology of the isolated exosomes was examined by TEM. WB was used to detect the exosome-positive markers (CD63, CD81, and Alix) and the exosome-negative marker calnexin.

The size distribution and particle concentrations of isolated N-EXO and AI-EXO were measured by nanoparticle tracking analysis (NTA). Briefly, exosome samples were diluted in particle-free PBS to an optimal detection concentration of 1 × 10^8^ particles/mL. Samples were then analyzed using a Particle Metrix ZetaView nanoparticle tracking analyzer (Particle Metrix GmbH, Bavaria, Germany), which tracked the Brownian motion of individual nanoparticles to calculate the particle concentration and size distribution.

The particle-to-protein ratio (PPR) was calculated as a standardized measure of exosome purity. Exosome particle concentration was determined by NTA (ZetaView), and the total protein content of matched samples was quantified using a bicinchoninic acid assay. The PPR was expressed as the number of particles per microgram of total protein (particles/μg protein).

For the cellular uptake assay, RAW264.7 cells were seeded in 12-well plates at a density of 1 × 10^6^ cells per well at 37 °C for 24 h. The cell culture medium was replaced with 1 mL of fresh medium containing DiD-labeled AI-EXO or N-EXO (5 × 10^8^ particles/mL). After 2 h of incubation, the cells were washed 3 times with PBS, fixed, counterstained with DAPI, and imaged using a laser-scanning confocal microscope (LSM 800, Zeiss, Germany).

To investigate proteomic variations between AI-EXO and N-EXO, we conducted label-free liquid chromatography-tandem mass spectrometry (LC-MS/MS)-based proteomic profiling. Differential protein expression was analyzed using the limma package (R version 4.3.1, Bioconductor framework). The Benjamini–Hochberg procedure was applied to correct for multiple testing and control the false discovery rate (FDR). Proteins with a fold change > 1.5 (upregulated) or < 0.667 (downregulated) and an adjusted P value < 0.05 were defined as differentially expressed proteins (DEPs). Gene Ontology (GO) and Kyoto Encyclopedia of Genes and Genomes (KEGG) enrichment analyses of the DEPs were performed using the clusterProfiler package. Enrichment results were visualized using ggplot2, with color gradients representing adjusted P values.

### 2.11 Analysis of M1 and M2 macrophage phenotype polarization

To establish reference macrophage phenotypes, RAW264.7 cells were stimulated with LPS (10 μg/mL) or IL-4 (20 ng/mL) for 24 h to induce the M1 (classically activated) or M2 (alternatively activated) phenotype, respectively.

The M1-to-M2 phenotypic transition was induced by treatment with AI-EXO (5 × 10^8^ particles/mL) or N-EXO (5 × 10^8^ particles/mL) for 24 h. Flow cytometry, immunofluorescence staining, western blotting, and qRT-PCR were used to quantify the M1 and M2 macrophage populations.

For flow cytometric analysis of macrophage polarization, RAW264.7 cells were collected, fixed, permeabilized, and stained with antibodies against F4/80 (1:200, Thermo Fisher Scientific, eBioscience, CA, USA), iNOS (M1 marker, 1:400, BioLegend, CA, USA), and Arg-1 (M2 marker, 1:400, Invitrogen, CA, USA). Gating was performed sequentially. Cells were first separated from debris based on forward and side scatter, after which singlets were gated using FSC-A versus FSC-H to exclude doublets. F4/80^+^ macrophages were then gated for subsequent analysis. The M2/M1 ratio was defined as the percentage of Arg-1^+^ M2 macrophages divided by the percentage of iNOS^+^ M1 macrophages. The complete gating strategy is provided in Supplementary Fig. S2.

For immunofluorescence staining, cells were fixed with paraformaldehyde, permeabilized with 0.1% Triton X-100, and then blocked with 10% serum for 45 min at 25 °C. Samples were then incubated with APC-conjugated anti-mouse iNOS (1:200, Affinity Biosciences, Jiangsu, China) and FITC-conjugated anti-mouse Arg-1 (1:200, Affinity Biosciences, Jiangsu, China) primary antibodies for 1 h at 37 °C. After DAPI nuclear counterstaining, images were obtained using a laser-scanning confocal microscope (LSM 880, Zeiss, Germany).

Western blotting was performed as described above. Briefly, the primary antibodies were anti-mouse iNOS (1:500, Affinity Biosciences, Jiangsu, China) and anti-mouse Arg-1 (1:500, Affinity Biosciences, Jiangsu, China). Goat anti-rabbit IgG (H + L) (1:10,000; Affinity Biosciences, Jiangsu, China) was used as the secondary antibody.

qRT-PCR was performed using the primers listed in Supplementary Table S1. Relative gene expression was calculated using the 2−ΔΔCt method, with GAPDH as the reference gene and the blank control as the calibrator. All procedures were performed according to the manufacturer's instructions.

### 2.12 Establishment of a diabetic wound healing model in rats

Male Sprague-Dawley rats (250-300 g) were purchased from the Animal Center of Southern Medical University. All procedures were approved by the Southern Medical University Ethics Committee (Guangzhou, China; approval no. LAEC-2023-044) and were performed in accordance with National Institutes of Health guidelines for the care and use of laboratory animals. Diabetes was induced by streptozotocin (STZ) injection combined with a high-sugar, high-fat diet according to an established protocol [33]. Briefly, all rats were fed with a high-sugar, high-fat diet for 4 weeks. Beginning in week 5, rats received intraperitoneal injections of STZ (30 mg/kg in citrate buffer, pH 4.2-4.5) for 5 consecutive days after a 12-h fast and remained on the same diet. Tail-vein blood glucose was measured every 3 days. Rats were considered diabetic if fasting blood glucose remained above 16.7 mmol/L for 2 weeks after the first STZ injection (Fig. S3A). After anesthesia, a 15-mm full-thickness skin wound was created on the back of each rat. The rats were then randomly assigned to six groups: control (120 μL PBS applied locally); tail vein + PBS (120 μL PBS injected via the tail vein); tail vein + AI (120 μL of 1 mM AICAR injected via the tail vein); MEWs + GelMA; MEWs + GelMA@AI; and MEWs + GelMA@AI + MER-i (120 μL of 1 mM AICAR plus 1 μM UNC2881). To prevent wound contraction, silicone rings were sutured around the wound margins. Digital photographs were obtained on post-wounding days 0, 3, 7, and 14, and wound areas were quantified using ImageJ.

### 2.13 Histological analysis

Wound specimens from diabetic rats were collected on day 3, 7, and 14 post-wounding, fixed with 4% paraformaldehyde, and processed into 4 μm paraffin sections. For histological examination, sections were deparaffinized, rehydrated, and stained with hematoxylin and eosin (H&E) or Masson's trichrome. Angiogenesis was assessed by immunohistochemical staining for CD31. Briefly, tissue sections underwent antigen retrieval in sodium citrate for 15 min and were then blocked with 3% BSA (Solarbio, China) for 30 min. Sections were incubated overnight at 4 °C with an anti-CD31 antibody (1:100; Abcam), followed the next day by an HRP-conjugated secondary antibody for 50 min and hematoxylin and eosin staining. Images were acquired using an upright optical microscope and analyzed with ImageJ. For immunofluorescence staining, tissue sections underwent antigen retrieval and 10-min permeabilization, followed by blocking with 10% goat serum to minimize nonspecific binding. Primary antibodies against iNOS (1:500; Novus, Germany) and Arg-1 (1:200; Affinity Biosciences, China), together with the corresponding secondary antibodies, were used to evaluate macrophage phenotypes in wound tissues. Nuclei were stained with DAPI for 5 min. Immunofluorescence images were acquired by laser-scanning confocal microscopy (LSM 880, Zeiss, Jena, Germany) and quantified using ImageJ.

### 2.14 Statistical analyses

Data are presented as the mean ± standard deviation (SD) from at least 3 independent experiments. All n values denote the number of technical replicates in each experiment. Normality and homogeneity of variance were assessed using the Shapiro-Wilk and Brown-Forsythe tests, respectively, before parametric analyses. Two groups were compared using an unpaired, two-tailed Student's *t*-test when the assumptions for parametric testing were met. Multiple groups were compared using one-way analysis of variance followed by Tukey's post hoc test to adjust for multiple pairwise comparisons. A two-sided P value < 0.05 was considered statistically significant. Significance levels are indicated as *P < 0.05 and **P < 0.01.

## 3 Results

### 3.1 AICAR activated AMPK to enhance the energy supply of HUVECs under inflammatory conditions

#### 3.1.1 AICAR activated AMPK

Tissue repair efficiency is tightly regulated by the homeostatic balance of cellular energy metabolism, in which adequate ATP production is essential for the biosynthetic and functional activities of regenerating cells [34], [35]. AMPK is regarded as a central metabolic switch [36], [37]. In our previous study, AICAR-induced AMPK phosphorylation enhanced EC energy metabolism, thereby improving the structural and functional maturation of small-diameter tissue-engineered vascular grafts [18]. In this study, HUVECs were primed with LPS for 24 h to model the inflammatory conditions of diabetic wounds *in vitro*, LPS pretreatment was used in all subsequent *in vitro* cell experiments. WB analysis revealed a 3.25 ± 0.62-fold increase in AMPK phosphorylation after AICAR treatment compared with untreated controls, whereas cotreatment with the AMPK inhibitor, Compound C, abolished this activation (Fig. 1A).

**Fig. 1:**
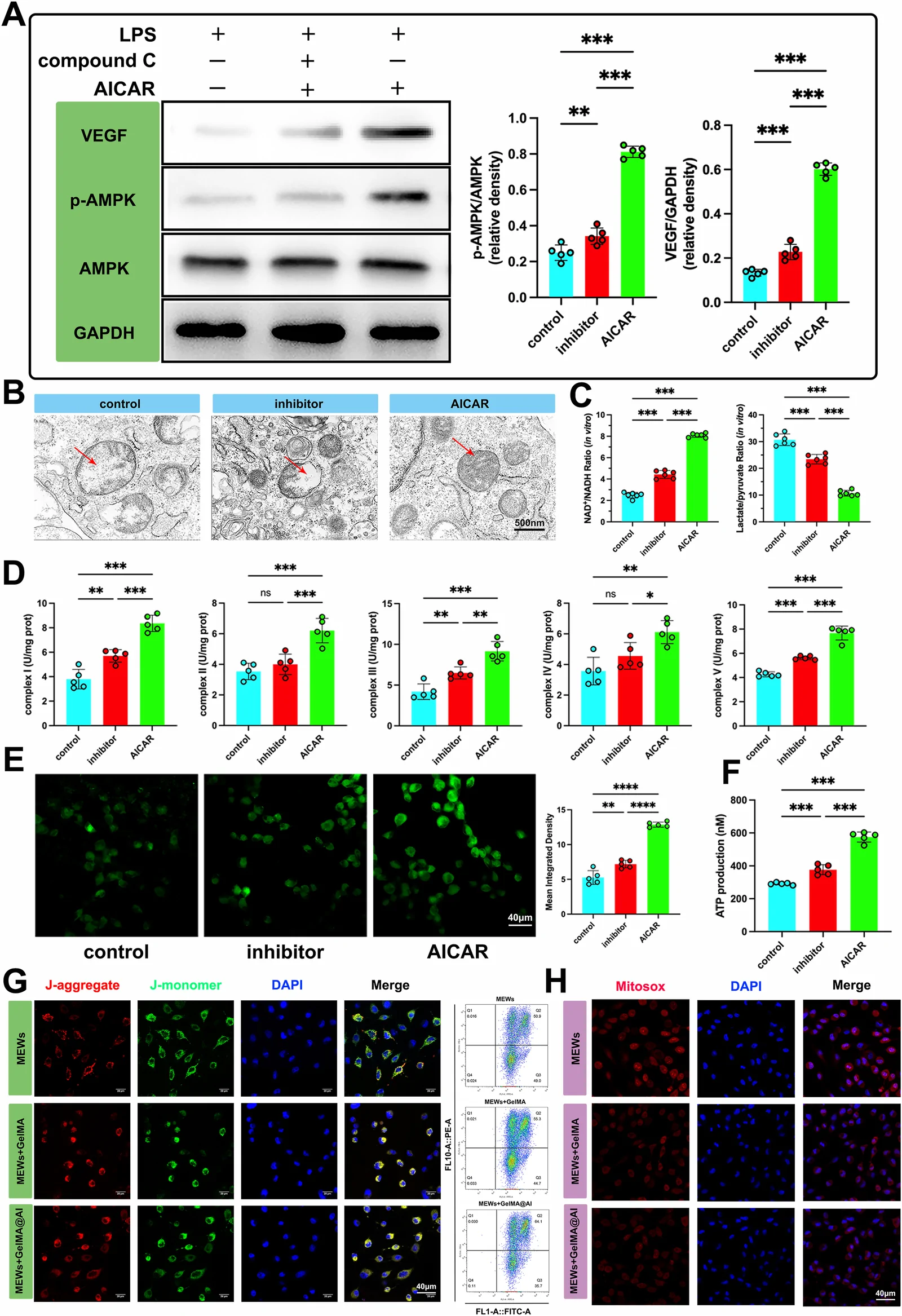
AICAR activated AMPK and enhanced the energy supply of HUVECs under inflammatory conditions. A) AICAR activated AMPK in LPS-treated HUVECs (n = 5). B) TEM images showing mitochondrial ultrastructure in HUVECs (representative mitochondria, red arrows; scale bar = 500 nm). C) NAD^+^/NADH and lactate/pyruvate ratios. D) Activities of mitochondrial respiratory-chain complexes after AICAR treatment (n = 5). E) MitoTracker Green staining showing increased mitochondrial mass after AICAR treatment (scale bar = 40 μm; n = 5). F) ATP production (n = 5). G) JC-1 staining showing an increase in mitochondrial membrane potential after AICAR treatment (scale bar = 40 μm). H) MitoSOX Red staining showing reduced mitochondrial superoxide levels after AICAR treatment (scale bar = 40 μm). Data are presented as the mean ± SD from three independent experiments. Multiple groups were compared using one-way ANOVA followed by Tukey's post hoc test. *P < 0.05, **P < 0.01, and ***P < 0.001. LPS and Compound C were used at 10 μg/mL and 10 μM, respectively.

#### 3.1.2 AICAR enhanced the energy supply of HUVECs

Mitochondria play multifaceted roles in tissue regeneration by dynamically coordinating energy metabolism, redox signaling, and cellular plasticity [38], [39], [40], [41]. To determine whether AICAR preserved mitochondria and improved mitochondrial function, we examined mitochondrial structure and function in HUVECs. TEM showed preserved mitochondrial ultrastructure in AICAR-treated cells, in contrast to the marked swelling and pyknotic morphology observed in the control and inhibitor groups (Fig. 1B). Quantitative MitoTracker Green fluorescence confirmed an increase in mitochondrial mass in the AICAR group (Fig. 1E). Functional assays revealed that AICAR significantly increased the activities of respiratory-chain complexes I-V (Fig. 1D), accompanied by a 2.0-fold increase in ATP production relative to the control group (Fig. 1F). Mitochondrial membrane potential (MMP) was measured using the JC-1 probe. Fluorescence imaging and flow cytometry showed that AICAR significantly increased the mitochondrial membrane potential (Fig. 1G and Fig. S4A). MitoSOX Red staining further showed that AICAR reduced mitochondrial superoxide levels (Fig. 1H and Fig. S4B). Collectively, these findings indicate that AICAR preserves mitochondrial integrity and enhances cellular energy supply.

To further validate the AICAR-induced high-energy state, we measured key metabolic redox indicators. AICAR treatment significantly elevated the NAD^+^/NADH ratio and reduced the lactate/pyruvate ratio in LPS-primed HUVECs, and both effects were abolished by AMPK inhibition with Compound C (Fig. 1C). These results demonstrate that AICAR shifts endothelial metabolism toward enhanced oxidative phosphorylation and suppressed glycolysis in an AMPK-dependent manner, supporting the establishment of a highly efficient, high-energy phenotype.

### 3.2 AICAR enhanced the angiogenic capacity of HUVECs *in vitro*

Angiogenesis plays a key role in diabetic wound healing [42], [43], [44]. Newly formed microvessels support wound repair by delivering oxygen and nutrients and removing metabolic waste. Western blotting showed a marked increase in VEGF expression after AICAR treatment compared with untreated controls (Fig. 1A). Immunofluorescence staining of VEGF protein further confirmed that AICAR could enhance the secretion of VEGF (Fig. 2A and B). Scratch-wound, chick chorioallantoic membrane, and tube-formation assays demonstrated that AICAR enhanced the angiogenic capacity of HUVECs *in vitro* (Fig. 2C–G), whereas Compound C suppressed these proangiogenic effects. These findings indicate that AICAR has therapeutic potential for promoting diabetic wound healing by enhancing angiogenesis.

**Fig. 2:**
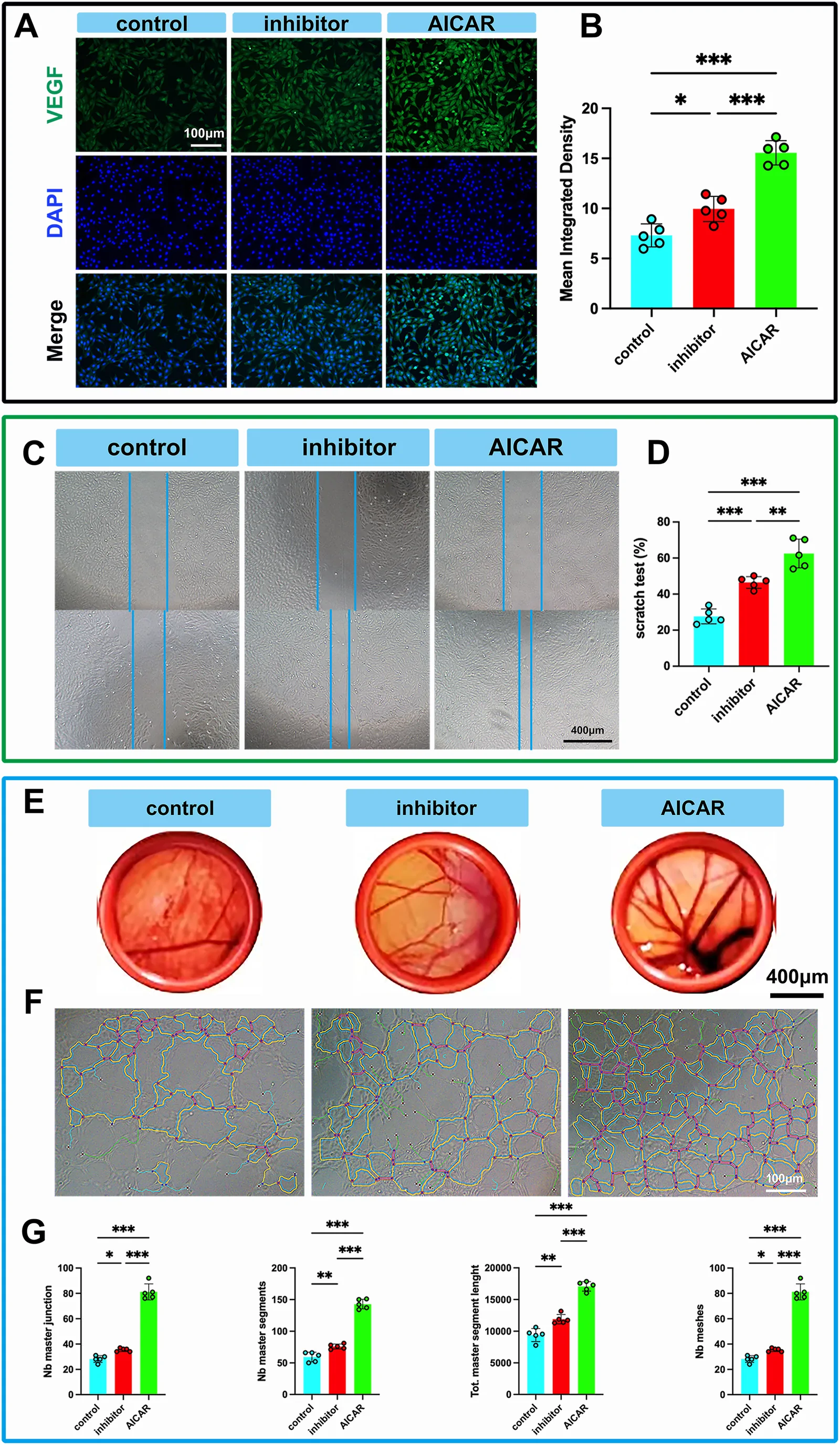
AICAR enhanced the angiogenic capacity of HUVECs *in vitro*. A, B) VEGF expression after AICAR treatment (scale bar = 100 μm; n = 5). C, D) Migration of HUVECs after AICAR treatment (scale bar = 400 μm; n = 5). E) Chick chorioallantoic membrane assay showing enhanced angiogenic capacity after AICAR treatment (scale bar = 100 μm). F, G) Tube-formation assay and quantitative analysis showing enhanced angiogenic capacity after AICAR treatment (scale bar = 100 μm; n = 5). Data are presented as the mean ± SD from three independent experiments. Multiple groups were compared using one-way ANOVA followed by Tukey's post hoc test. *P < 0.05, **P < 0.01, ***P < 0.001.

### 3.3 Characterization of AI-EXO

Exosomes are nanosized extracellular vesicles that transport bioactive cargo and mediate intercellular communication during tissue regeneration [45], [46], [47]. We demonstrated that AICAR enhanced EC energy status and angiogenic capacity; however, whether AICAR-preconditioned high-energy ECs regulate diabetic wound healing through exosome secretion remained unknown. HUVECs were therefore treated with AICAR to induce a high-energy state, after which exosomes were isolated from the culture supernatant, as illustrated in Fig. 3A. TEM revealed vesicles with the characteristic cup-shaped morphology of exosomes, and the size distributions are shown in Fig. 3B. The identity and purity of the isolated exosomes were validated by marker profiling. All three canonical exosome-positive markers (CD63, CD81, and protein Alix) were clearly detected in both N-EXO and AI-EXO samples whereas calnexin was undetectable in either preparation (Fig. 3C). The PPRs were 3.69 × 10^10^ particles/μg protein for N-EXO and 3.96 × 10^10^ particles/μg protein for AI-EXO (Fig. 3D). Both values were within the accepted range for high-purity exosome preparations and did not differ significantly between groups. To determine whether AICAR was encapsulated within AI-EXO, we performed LC-MS/MS. AICAR was below the limit of detection in purified AI-EXO lysates, indicating that the biological effects of AI-EXO were attributable to their intrinsic bioactive cargo rather than to encapsulated or residual free AICAR (Fig. S5A and B).

**Fig. 3:**
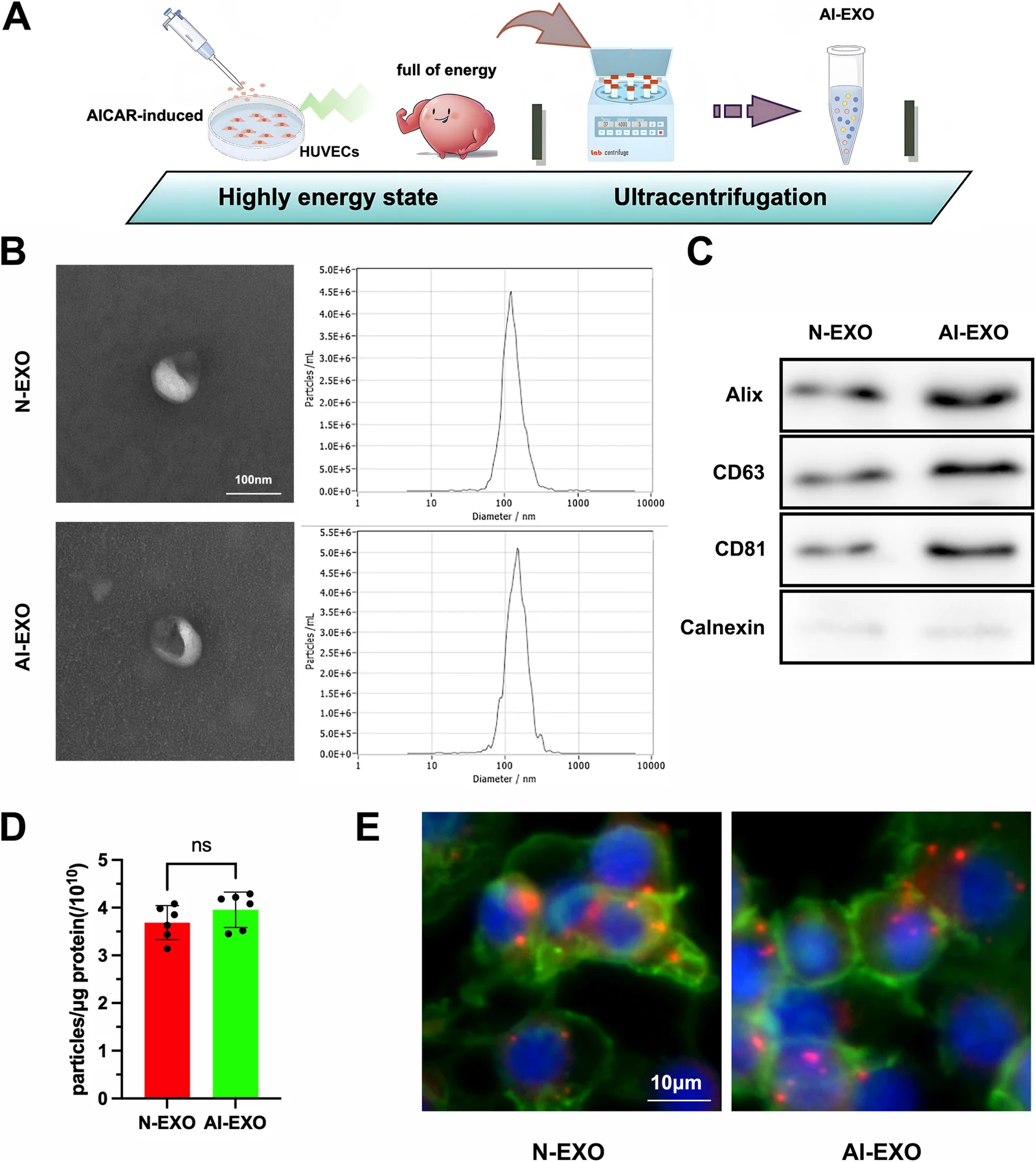
Characterization of AI-EXO. A) Schematic of the AI-EXO isolation procedure. B) TEM images showing the characteristic cup-shaped morphology of exosomes and particle size distribution (scale bar = 50 nm). C) Western blot analysis of exosome markers. D) Quantitative analysis of the particle-to-protein ratio. E) Uptake of labeled AI-EXO and N-EXO by GFP-labeled RAW264.7 cells. Data are presented as the mean ± SD from three independent experiments. Two groups were compared using an unpaired, two-tailed Student's *t*-test. ***P < 0.001.

AICAR stimulation significantly increased exosome production by HUVECs, with an approximately 3.0-fold higher AI-EXO yield than N-EXO yield. AI-EXO also contained 2.2-fold more bioactive protein than N-EXO (Fig. S6A and B). These results indicate that AICAR stimulation markedly enhanced exosome production without compromising the isolated purity. Given the pivotal role of macrophage polarization in regulating the inflammatory microenvironment and regenerative responses, we next evaluated exosome uptake by RAW264.7 macrophages. Both AI-EXO and N-EXO were efficiently internalized (Fig. 3E), suggesting that AI-EXO may influence diabetic wound healing by modulating macrophage polarization.

### 3.4 AI-EXO induced macrophage phenotypic reprogramming via GAS6 protein *in vitro*

#### 3.4.1 Proteomic analysis of N-EXO and AI-EXO

Recent advances in extracellular vesicle research have established exosomes as important mediators of tissue homeostasis [48]. Their bioactive cargo enables intercellular communication that is increasingly recognized as essential for successful tissue repair. Quantitative analyses showed that AI-EXO had a 3.0-fold higher vesicle yield and 2.2-fold greater bioactive protein content than N-EXO. We therefore hypothesized that AICAR enhances HUVEC angiogenesis and other proregenerative functions through bioactive exosomal cargo. To investigate this mechanism, we performed comparative proteomic profiling of AI-EXO and N-EXO, followed by GO and KEGG enrichment analyses of differentially expressed proteins. KEGG analysis identified differentially expressed proteins associated with energy-metabolism pathways, including fatty acid metabolism (blue arrows), oxidative phosphorylation (blue arrows), and the citrate cycle (Fig. S7A and B). Our previous study showed that AICAR enhanced tissue regeneration while increasing M2 macrophage polarization [18]. In addition, increased oxidative metabolism and energy production are widely considered to promote M2 polarization and tissue repair. We therefore hypothesized that AI-EXO induces M2 macrophage polarization. The heatmap showed distinct proteomic profiles in the two groups, with growth arrest-specific protein 6 (GAS6) among the most significantly upregulated proteins in AI-EXO (Fig. 4B, red arrow). The complete list of differentially expressed proteins is provided in the Supplementary Materials. A schematic of AI-EXO-induced M1-to-M2 macrophage reprogramming through the GAS6/MER/AKT signaling pathway is shown in Fig. 4A.

**Fig. 4:**
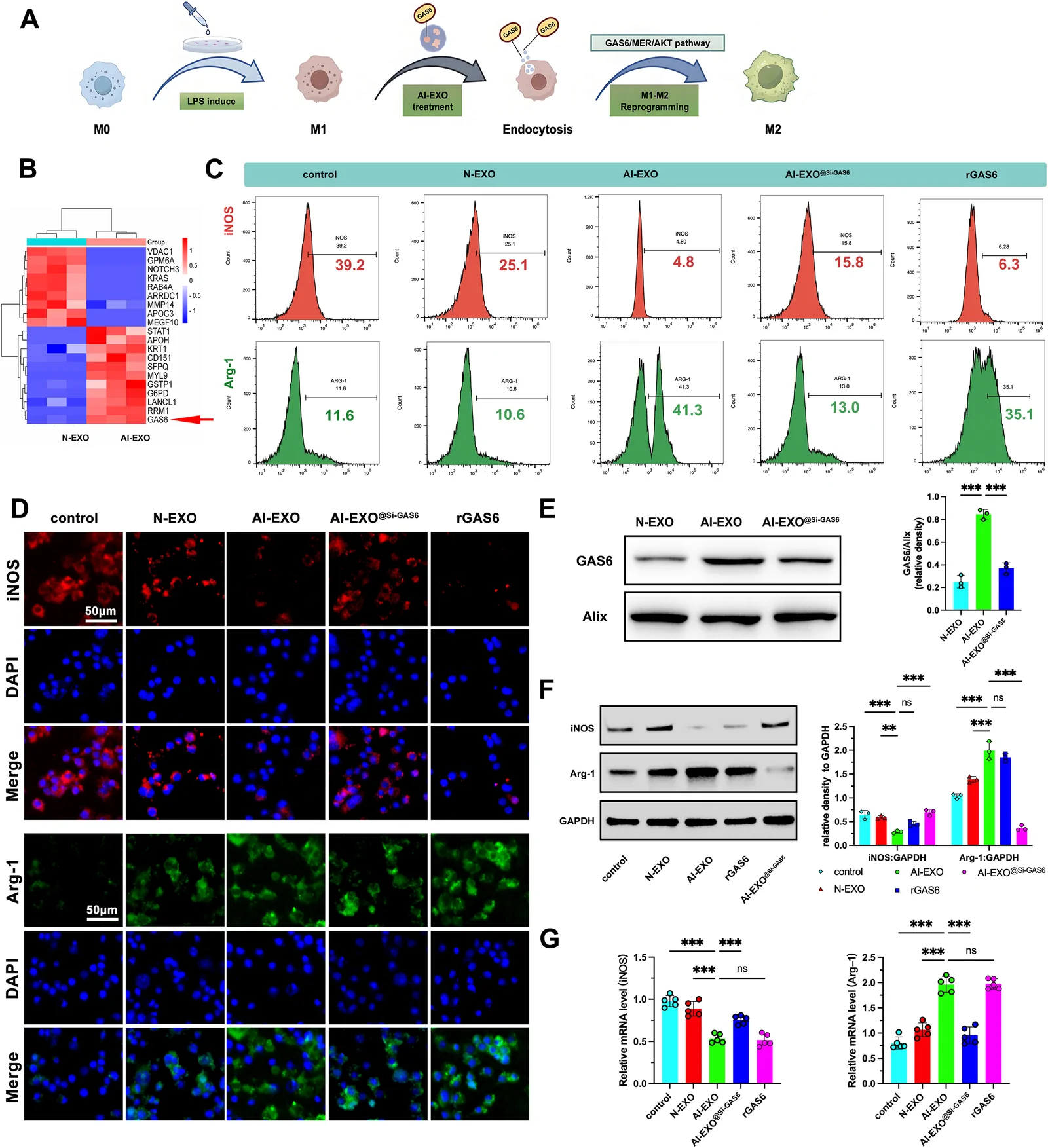
AI-EXO induced macrophage phenotypic reprogramming via GAS6 protein *in vitro*. A) Schematic of AI-EXO-induced M1-to-M2 macrophage reprogramming through GAS6/MER/AKT signaling pathway. B) Proteomic analysis of N-EXO and AI-EXO. Heatmap analysis showed significant enrichment of growth arrest-specific protein 6 (GAS6; red arrow). C, D) AI-EXO reprogrammed proinflammatory M1 macrophages to an anti-inflammatory M2 phenotype. RAW264.7 cells were pre-stimulated with LPS to induce M1 polarization or with IL-4 to induce M2 polarization. C) Flow cytometry plots showing the percentages of iNOS^+^ M1 and Arg-1^+^ M2 macrophages in each group. D) Immunofluorescence staining of iNOS (M1 marker; red), Arg-1 (M2 marker; green), and nuclei (blue) in M1 macrophages after the indicated treatments (scale bar = 50 μm). E) Western blot analysis and quantification of GAS6 protein levels in N-EXO and AI-EXO (n = 3). F) Western blot analysis and quantification of iNOS and Arg-1 protein levels in the indicated groups (n = 3). G) Quantification of iNOS and Arg-1 mRNA expression (n = 3). Data are presented as the mean ± SD from three independent experiments. Multiple groups were compared using one-way ANOVA followed by Tukey's post hoc test; two groups were compared using an unpaired, two-tailed Student's *t*-test. *P < 0.05, **P < 0.01, and ***P < 0.001.

#### 3.4.2 AI-EXO reprogrammed proinflammatory M1 macrophages to anti-inflammatory M2 phenotype

GAS6 is a secreted immunomodulatory ligand with potent anti-inflammatory and tissue-protective effects in preclinical disease models [49], [50]. Clinically, serum GAS6 levels are significantly reduced in patients with rheumatoid arthritis (RA) compared with healthy donors, with the lowest concentrations observed in patients with erosive RA [51]. Macrophage profiling has shown that MER is downregulated in proinflammatory M1 macrophages from patients with RA and healthy donors, whereas anti-inflammatory M2 macrophages exhibit increased MER expression [52], [53]. Together, these observations suggest that GAS6 carried by AI-EXO may reshape macrophage function. To determine the role of GAS6 in AI-EXO-mediated M1-to-M2 reprogramming, GAS6 was knocked down in HUVECs before induction of the high-energy state with AICAR. The resulting exosomes, which expressed low levels of GAS6, were designated AI-EXO^@si−GAS6^ and used in subsequent experiments. Western blotting showed that si-GAS6-3 produced the most efficient GAS6 knockdown among the three siRNAs (Fig. S8A and B), and GAS6 levels in the exosomes were subsequently confirmed by western blotting (Fig. 4E).

The effects of AI-EXO on macrophage reprogramming were evaluated by immunofluorescence staining, western blotting, flow cytometry, and qRT-PCR. Flow cytometry showed that AI-EXO induced robust M1-to-M2 reprogramming. The proportion of iNOS^+^ M1 macrophages decreased to 4.8%, whereas that of Arg-1^+^ macrophages increased to 41.3%, yielding an M2/M1 ratio of approximately 8.60, compared with approximately 0.30 in the control group and 0.42 in the N-EXO group (Fig. 4C). GAS6 knockdown in AI-EXO abrogated this effect, restoring the proportions of iNOS^+^ (15.8%) and Arg-1^+^ (13.0%) macrophages to levels comparable to those in the N-EXO group. To further confirm the causal role of GAS6 and exclude potential off-target effects of siRNA-mediated knockdown, we performed rescue experiments by supplementing AI-EXO@si-GAS6 with recombinant GAS6 (rGAS6). rGAS6 supplementation restored the macrophage reprogramming effect and recapitulated the phenotype induced by AI-EXO: the proportion of iNOS^+^ M1 macrophages was reduced to 6.3%, whereas that of Arg-1^+^ M2 macrophages was increased to 35.1%, yielding an M2/M1 ratio of approximately 5.59. Immunofluorescence staining, WB, and qRT-PCR yielded consistent findings (Fig. 4D–F, G). AI-EXO also increased the proportion of M2-phenotype macrophages among IL-4-induced RAW264.7 cells (Fig. S9A and B). Collectively, these findings identify GAS6 as a critical mediator of M1-to-M2 macrophage phenotypic reprogramming.

### 3.5 AI-EXO enhanced M1-to-M2 reprogramming by delivering GAS6 and activating the MER/AKT pathway *in vitro*

Activation of TAM receptors represents a promising anti-inflammatory therapeutic strategy, and MER is a high-affinity receptor for GAS6 [49], [54], [55]. In our study, we first treated LPS-induced M1 macrophages with the indicated exosome preparations and then assessed AKT and p-AKT expression by western blotting. p-AKT expression increased in the AI-EXO group but remained similar to control levels in the AI-EXO@si-GAS6, N-EXO, and untreated control groups. Supplementation of AI-EXO@si-GAS6 with rGAS6 reproduced the effects of AI-EXO (Fig. 5A). qRT-PCR and flow cytometry were used to verify the role of AKT phosphorylation in GAS6-regulated M1-to-M2 reprogramming. The M2/M1 ratio increased significantly in LPS-induced M1 macrophages after AI-EXO treatment (approximately 8.08 in the AI-EXO group vs. 0.35 in the control group and 0.45 in the N-EXO group), and this effect was abolished by the AKT inhibitors perifosine (5 μM) and MK-2206 (5 μM) (Fig. 5B–D). Comparable results were obtained with the MER inhibitors UNC2881 (1 μM) and UNC2250 (1 μM) (Fig. 5E–G). Together, these findings indicate that AI-EXO promotes M1-to-M2 macrophage reprogramming by delivering GAS6 and activating the MER/AKT pathway. To further corroborate the specificity of the GAS6/MER signaling axis and rule out potential off-target artifacts of pharmacological receptor inhibition, we performed *in vitro* ligand neutralization assays using a GAS6-specific neutralizing antibody (2 μg/mL, GAS6 nAb, MAB885, R&D Systems, MN, USA). Pre-incubation of AI-EXO with GAS6 nAb significantly abrogated the M2 polarization effect induced by AI-EXO in LPS-primed RAW264.7 macrophages. Concordant alterations in iNOS (M1 marker) and Arg-1 (M2 marker) expression were consistently observed across immunofluorescence staining, flow cytometry, and western blot analyses (Fig. S10A–C). These data provide independent ligand-level evidence that the immunomodulatory function of AI-EXO is specifically mediated by exosomal GAS6, rather than non-specific off-target effects of small-molecule receptor inhibitors.

**Fig. 5:**
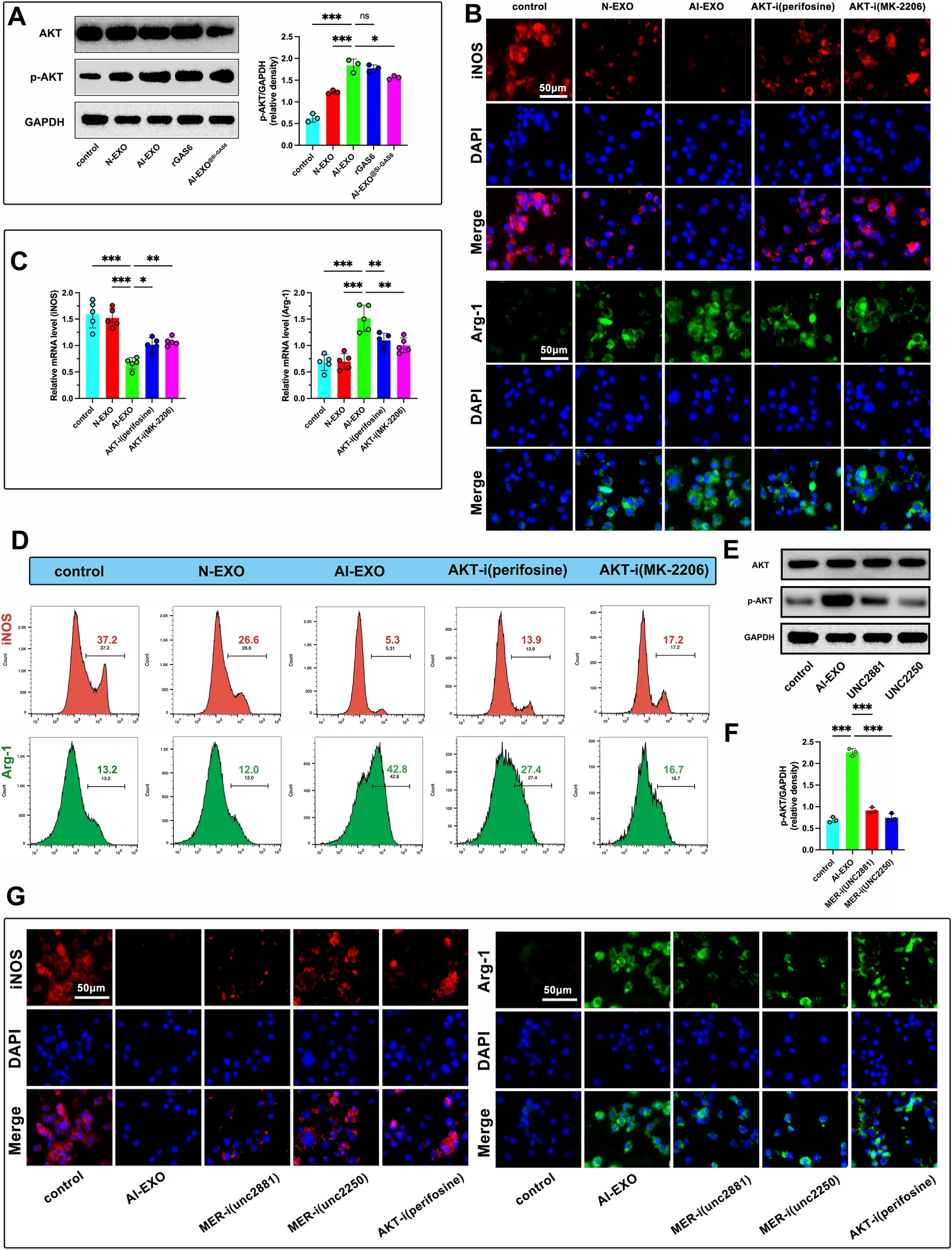
AI-EXO enhanced M1-to-M2 reprogramming by delivering GAS6 and activating the MER/AKT pathway *in vitro*. A) GAS6 carried by AI-EXO increased p-AKT expression in M1 macrophages (n = 3). B-D) Immunofluorescence staining (B), quantification of mRNA expression (C), and flow cytometry results (D) showing that the AKT inhibitor perifosine, attenuated the AI-EXO-mediated M1-to-M2 reprogramming (scale bar = 50 μm, n = 3). E-F) Western blot analysis and quantification showing that the MER inhibitor UNC2881 reduced AI-EXO-induced AKT phosphorylation (n = 3). G) Immunofluorescence staining showing that AKT or MER inhibition attenuated AI-EXO-mediated M1-to-M2 reprogramming (scale bar = 50 μm). Data are presented as the mean ± SD from three independent experiments. Multiple groups were compared using one-way ANOVA followed by Tukey's post hoc test; two groups were compared using an unpaired, two-tailed Student's *t*-test. *P < 0.05, **P < 0.01, and ***P < 0.001.

### 3.6 Characterization of scaffolds and visualization of HUVEC attachment and expansion *in vitro*

Persistent local immune dysregulation and excessive inflammation are major causes of chronic nonhealing diabetic wounds [56], [57]. To validate the potential of AICAR to promote diabetic wound healing, we fabricated a melt electrospinning writing PCL scaffold loaded with AICAR (MEWs + GelMA@AI). The fabrication process and gross and microscopic surface morphologies are shown in Fig. 6A and B. The AICAR release profile of MEWs + GelMA@AI is shown in Fig. 6C and demonstrates sustained release for approximately 15 days. Successful incorporation of AICAR into the scaffold was confirmed by FTIR spectroscopy (Fig. 6D). The compressive modulus of MEWs + GelMA@AI was 216.81 ± 10.11 kPa, compared with 148.89 ± 6.76 kPa for MEWs (Fig. S11A). This moderate increase in compressive modulus maintained scaffold integrity and supported stable AICAR release during wound healing. The equilibrium swelling ratio of MEWs + GelMA@AI was 4.81 ± 0.53%, significantly lower than that of pure GelMA (10.97 ± 1.22%) (Fig. S11B). The reduced swelling capacity limited excessive expansion and maintained stable adhesion to the wound surface. We further quantified the AICAR loading by measuring encapsulation efficiency and drug-loading content. The encapsulation efficiency of AICAR and the drug-loading content of the MEWs + GelMA@AI scaffolds were 77.68 ± 3.87% and 1.07 ± 0.07 μg/mg, respectively (Supplementary Table S2). Live/dead staining showed that HUVECs cultured on MEWs + GelMA@AI had a higher survival rate under inflammatory conditions (approximately 94.89%) than those cultured on MEWs (77.64%) or MEWs + GelMA (84.32%) (Fig. 6E and F). Phalloidin staining was used to visualize HUVEC growth on the scaffolds and showed the greatest cell attachment on MEWs + GelMA@AI (approximately 4145 cells), compared with MEWs (2334 cells) and MEWs + GelMA (2962 cells) (Fig. 6G and H). These results indicate that AICAR-loaded electrostatic direct-writing scaffolds are suitable for *in vivo* transplantation.

**Fig. 6:**
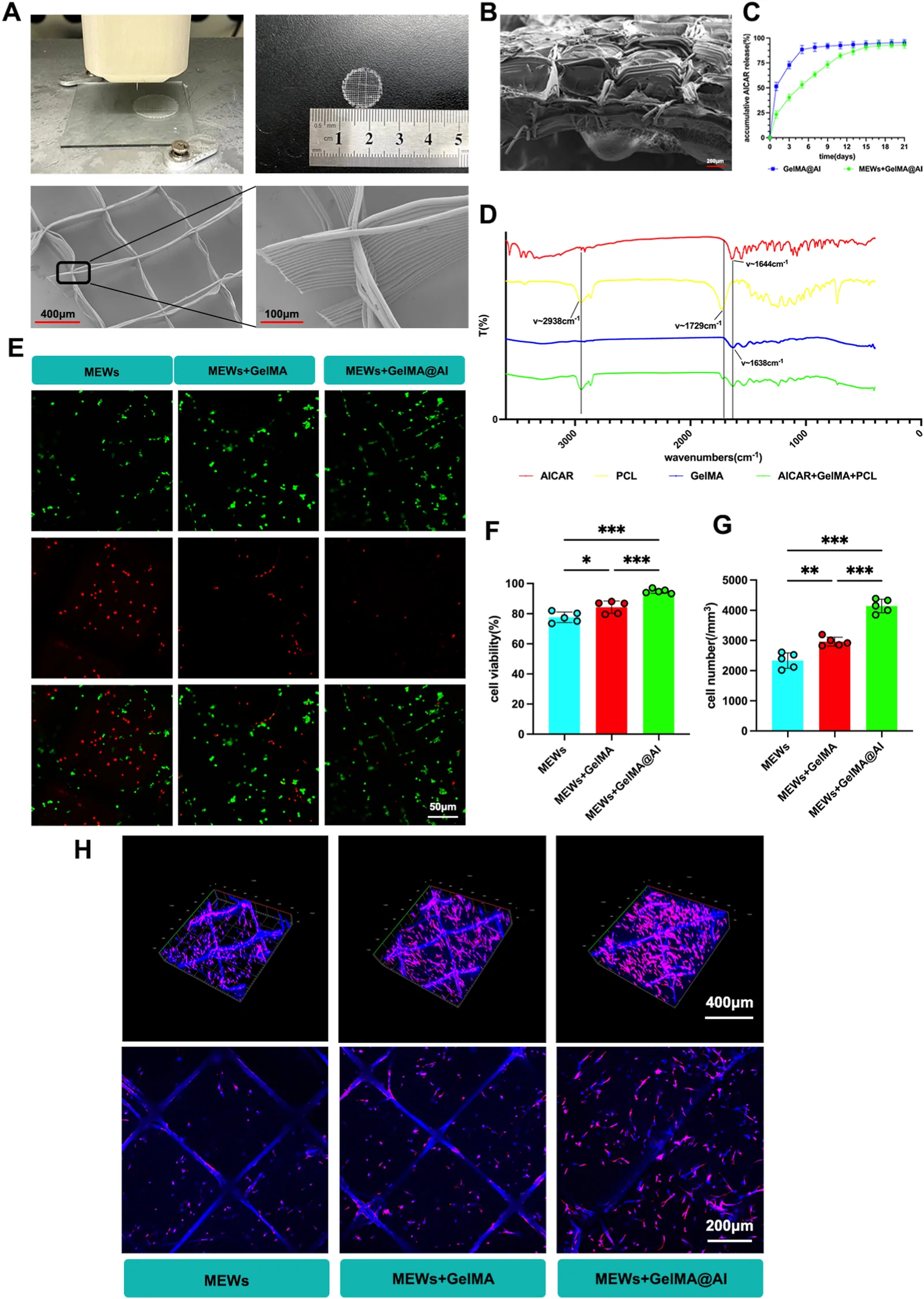
Characterization of MEWs scaffolds and visualization of HUVEC attachment and expansion across different MEWs groups *in vitro*. A) Schematic of MEWs scaffold fabrication and representative SEM images showing the scaffold surface morphology and porous microstructure at different magnifications (scale bars = 400 μm and 100 μm). B) SEM image showing the surface morphology of MEWs + GelMA@AI (scale bar = 200 μm). C) *In vitro* cumulative AICAR release from the GelMA@AI hydrogel and MEWs + GelMA@AI scaffold in PBS at 37 °C, demonstrating sustained release from the composite scaffold (n = 3). D) Fourier transform infrared (FTIR) spectra of pure AICAR, PCL, GelMA, and MEWs + GelMA@AI scaffold. E, F) Viability and quantitative analysis of HUVECs cultured on the MEWs (green, calcein AM-positive live cells; red, propidium iodide-positive dead cells; scale bar = 50 μm; n = 5). G, H) Confocal images (H) and quantitative analysis (G) of HUVEC attachment and expansion in MEWs scaffolds (scale bars = 400 and 200 μm). Data are presented as the mean ± SD from three independent experiments. Multiple groups were compared using one-way ANOVA followed by Tukey's post hoc test; two groups were compared using an unpaired, two-tailed Student's *t*-test. *P < 0.05, **P < 0.01, and ***P < 0.001.

### 3.7 Effects of MEWs + GelMA@AI on wound healing *in vivo*

To evaluate the effects of MEWs + GelMA@AI on chronic diabetic wounds, we transplanted the scaffolds onto wounds in diabetic rats. The complete *in vivo* experimental procedure is shown in Fig. 7A. Wounds were photographed on days 3, 7, and 14 after scaffold transplantation, and wound-closure rates were calculated at each time point. At each time point, rats were euthanized, and wound specimens were collected for H&E and Masson's trichrome staining. Compared with the other groups, the MEWs + GelMA@AI group exhibited faster wound closure (Fig. 7B and C), less inflammatory-cell infiltration on H&E staining, and greater collagen deposition on Masson's trichrome staining (Fig. 7, Fig. 8). Consistent with the *in vitro* findings, these histological improvements were markedly attenuated by MER inhibition in the MEWs + GelMA@AI + MER-i group, suggesting that the wound-healing effects of MEWs + GelMA@AI were mediated through the GAS6/MER/AKT pathway.

**Fig. 7:**
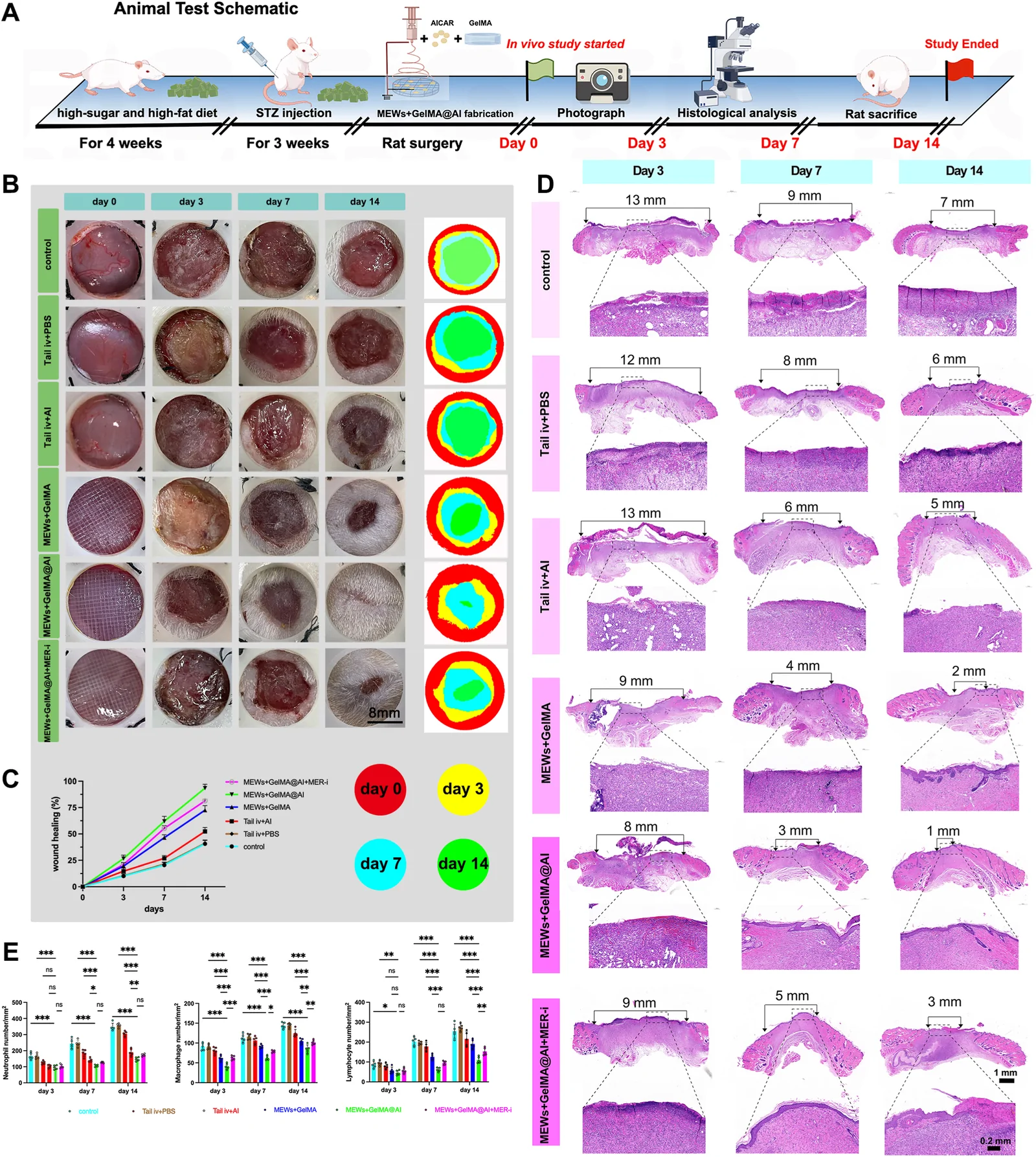
Effects of MEWs + GelMA@AI on wound healing *in vivo*. A) Schematic of the animal experiment. B) Representative gross images of wounds on days 0, 3, 7, and 14. C) Quantitative analysis of wound closure (n = 5). D) Representative H&E-stained wound sections. Unepithelialized areas are indicated by black arrows, and high-magnification views of wound tissues are delineated by dashed lines (scale bars = 1 and 0.2 mm). E) Quantification of inflammatory-cell infiltration (n = 5). Data are presented as the mean ± SD. ns, not significant. Multiple groups were compared using one-way ANOVA followed by Tukey's post hoc test. *P < 0.05, **P < 0.01, and ***P < 0.001.

**Fig. 8:**
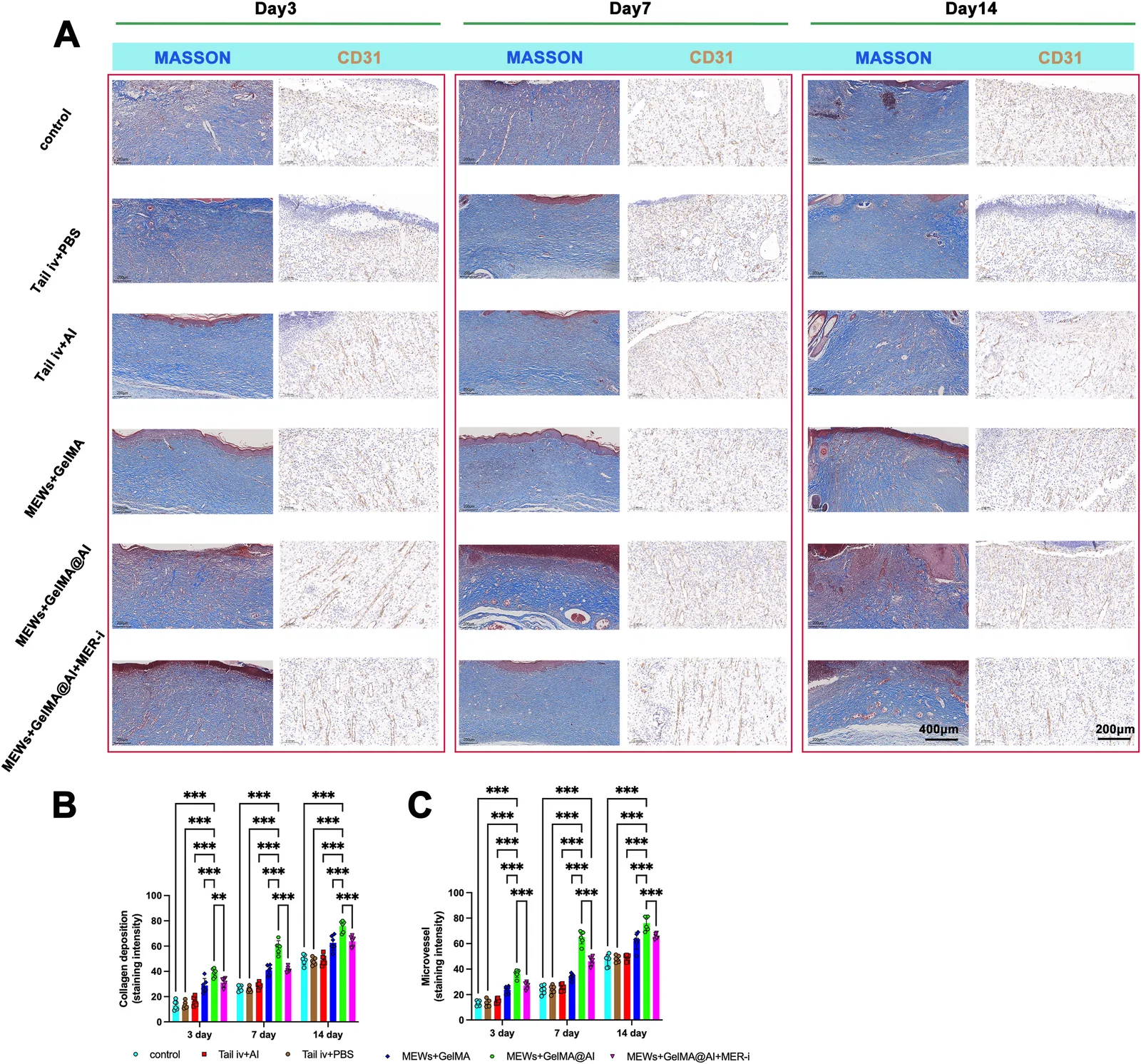
MEWs + GelMA@AI promoted collagen deposition and angiogenesis *in vivo*. A) Masson's trichrome staining of collagen fibers (blue) and CD31 immunohistochemistry for angiogenesis (scale bars = 200 and 400 μm). B, C) Quantitative analyses of collagen deposition and angiogenesis (n = 6). Data are presented as the mean ± SD. Multiple groups were compared using one-way ANOVA followed by Tukey's post hoc test. *P < 0.05, **P < 0.01, and ***P < 0.001.

To evaluate the systemic metabolic effects of scaffold-released AICAR, fasting blood glucose and body weight were measured in all rats throughout the experiment. Persistent hyperglycemia (fasting blood glucose > 16.7 mmol/L) was maintained in all diabetic rats from model induction to the study endpoint. No significant between-group differences in either measure was observed at any time point (Fig. S12A and B), indicating that neither local scaffold-mediated AICAR delivery nor systemic AICAR injection caused detectable systemic metabolic disturbances.

The biosafety of MEWs + GelMA@AI was evaluated by H&E staining of major organs. No significant histopathological differences were observed among groups, and no abnormalities were detected, indicating favorable biosafety of MEWs + GelMA@AI (Fig. S13). We further measured local glucose concentrations in wound tissues on days 3, 7, and 14. Diabetic wounds exhibited persistently higher glucose levels than those in normal wounds. MEWs + GelMA@AI treatment significantly reduced local glucose concentrations during the healing process, and this metabolic improvement was partially attenuated by MER inhibition (Fig. S3B), suggesting that the scaffold ameliorated the local hyperglycemic microenvironment in a GAS6/MER pathway-dependent manner.

### 3.8 MEWs + GelMA@AI promoted collagen deposition and angiogenesis *in vivo*

Angiogenesis plays a central role in diabetic wound healing [58], [59]. We used CD31 immunostaining to identify vascular ECs in wound tissues at each time point. AICAR significantly increased neovascularization *in vivo*, whereas less neovascularization was observed in the other groups (Fig. 8B and C).

### 3.9 MEWs + GelMA@AI promoted the M1-to-M2 phenotypic transition *in vivo*

Persistent excessive inflammation within the dysregulated immune microenvironment of chronic diabetic wounds substantially impedes healing [60]. We previously demonstrated *in vitro* that AICAR induced ECs to release high-energy exosomes (AI-EXO), which promoted M1-to-M2 macrophage reprogramming by delivering GAS6 and activating the MER/AKT signaling pathway. To validate whether AICAR produced similar effects *in vivo,* we first assessed the metabolic status of diabetic wound tissues. Consistent with the *in vitro* findings, the MEWs + GelMA@AI group exhibited a higher NAD^+^/NADH ratio and a lower lactate/pyruvate ratio than the control groups at all time points (Fig. S14A and B), indicating that local AICAR delivery ameliorated wound metabolic dysfunction. To confirm macrophage polarization *in vivo*, we performed F4/80 co-immunofluorescence staining of wound tissues on post-wounding day 14. Compared with the control groups, MEWs + GelMA@AI treatment significantly increased the proportion of F4/80^+^Arg-1^+^ M2 macrophages and decreased that of F4/80^+^iNOS^+^ M1 macrophages, consistent with the mRNA expression profiles (Fig. 9A–C). Notably, MER inhibition abolished this shift in macrophage polarization, demonstrating that the *in vivo* immunomodulatory effects of MEWs + GelMA@AI were mediated through GAS6/MER signaling.

**Fig. 9:**
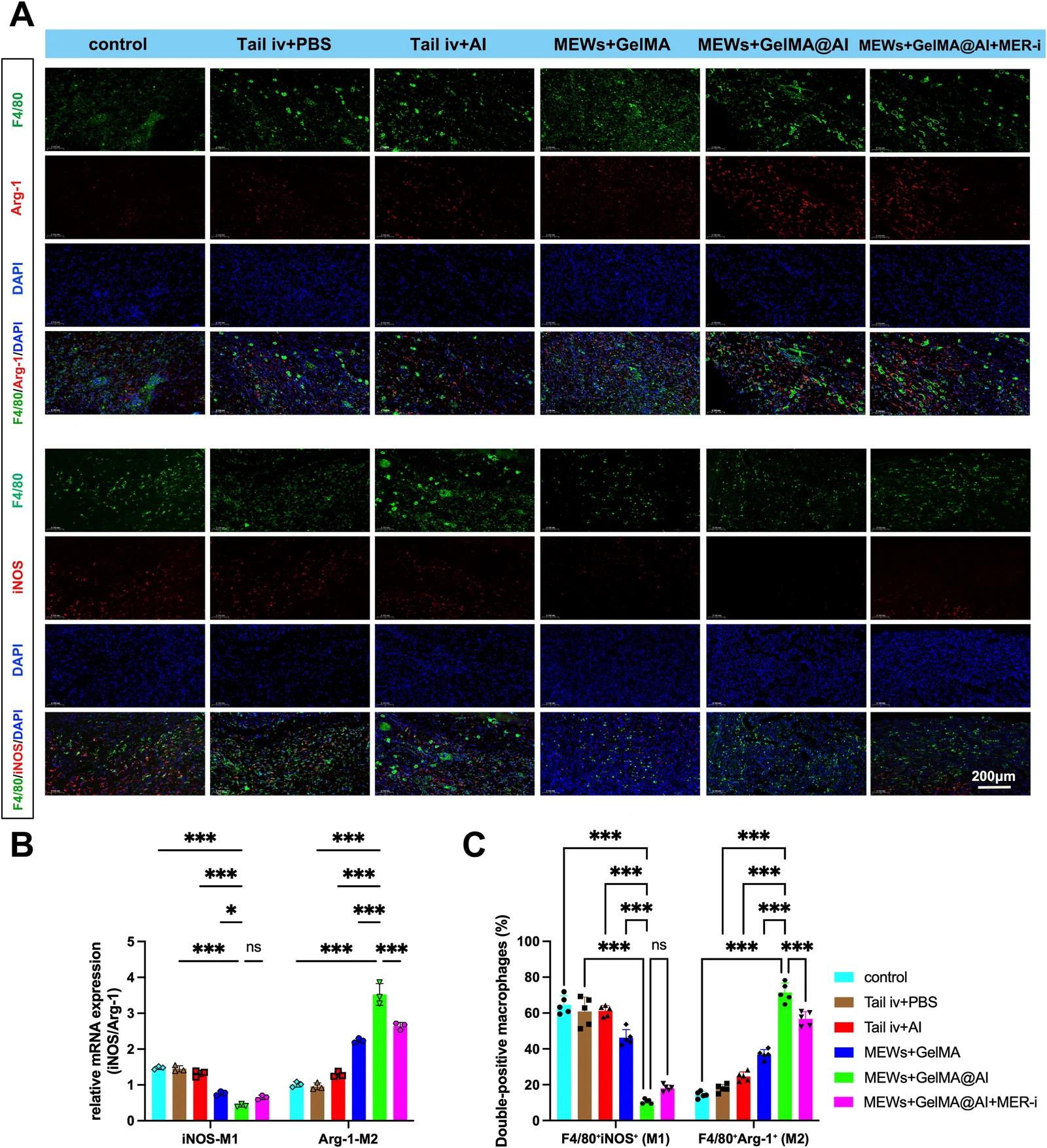
*In vivo* validation of macrophage polarization by F4/80 co-immunofluorescence on post-wounding day 14. A) Representative immunofluorescence images of wound sections co-labeled for the pan-macrophage marker F4/80 (green), M2 marker Arg-1 (red, upper panel), M1 marker iNOS (red, lower panel), and cell nuclei (DAPI, blue) (Scale bar = 200 μm). B) Relative mRNA expression of the M1 marker iNOS and M2 marker Arg-1 in wound tissues. C) Quantitative analysis of F4/80^+^iNOS^+^ M1 macrophages and F4/80^+^Arg-1^+^ M2 macrophages, expressed as percentages of total cells. Data are presented as the mean ± SD. Multiple groups were compared using one-way ANOVA followed by Tukey's post hoc test. *P < 0.05, **P < 0.01, and ***P < 0.001.

## 4 Discussion

Chronic diabetic wounds remain a substantial clinical burden, characterized by impaired angiogenesis and persistent pro-inflammatory macrophage activation, which collectively impede tissue repair [61], [62]. In this study, we demonstrate that AICAR-mediated AMPK activation reprograms endothelial cell metabolism, thereby enhancing angiogenic function and inducing the secretion of bioactive exosomes (AI-EXO). These exosomes drive M1-to-M2 macrophage polarization via exosomal GAS6 and the downstream MER/AKT signaling pathway. We further translated these mechanistic findings into a melt-electrospun PCL-GelMA scaffold (MEWs + GelMA@AI) that enables sustained local release of AICAR and accelerates diabetic wound healing *in vivo* without detectable systemic toxicity.

A key caveat of our exosome-related findings is the functional heterogeneity of extracellular vesicle populations. Our AI-EXO preparations, isolated by differential ultracentrifugation, comprise a heterogeneous mixture of small exosomes (30–150 nm) and larger microvesicles (150–1000 nm) (Fig. 3A and B). Mounting evidence indicates that distinct vesicle subpopulations possess unique cargo profiles and perform discrete biological functions in tissue homeostasis and repair [63], [64]. Therefore, GAS6 enrichment and the associated immunomodulatory activity are likely concentrated within a specific subset of small exosomes, rather than uniformly distributed across all secreted vesicles. High-resolution fractionation techniques, including iodixanol density-gradient ultracentrifugation and fluorescence-based nanoparticle flow cytometry, would facilitate the separation of these subpopulations, characterization of their respective cargo profiles, and more precise attribution of the observed pro-healing effects [65]. Such studies would further refine our mechanistic understanding and support the development of more targeted exosome-based therapeutics.

Our identification of exosomal GAS6 as a paracrine mediator of endothelial-macrophage crosstalk advances the current understanding of immunometabolic regulation in diabetic wound repair. Although the GAS6/MER/AKT axis has been implicated in anti-inflammatory signaling in other disease contexts [51], [54], [55], this study is the first to identify this pathway as a central link between endothelial metabolic status and macrophage phenotypic reprogramming in chronic wounds. From a translational perspective, this metabolic intervention strategy complements established and emerging wound therapies. For example, laser therapy accelerates wound closure and reduces scarring by modulating macrophage polarization and the expression of angiogenic growth factors through photo-biomodulation [66]. Similarly, mechanical tension-modulating approaches influence scar formation by regulating fibroblast activation and immune cell function through mechano-transduction pathways [67]. Consistent with the growing emphasis on multimodal wound care strategies, our approach provides a complementary, metabolism-centered modality that simultaneously targets both vascular insufficiency and immune dysregulation.

Several limitations of this study should be acknowledged. First, although the STZ-induced diabetic rat model recapitulates key features of hyperglycemia-associated wound-healing impairment, it does not fully reproduce the comorbidities, prolonged healing course, microbial colonization, and peripheral neuropathy characteristic of human diabetic ulcers [68]. Second, the *in vitro* mechanistic experiments relied on immortalized cell lines. Further validation using primary human endothelial cells and patient-derived macrophages would strengthen the physiological relevance and clinical translatability of the AMPK-GAS6 axis [69]. Third, although our 14-day *in vivo* study demonstrated favorable local and systemic biosafety, with no histopathological abnormalities detected in major organs, the long-term effects of sustained local AICAR release on tissue remodeling, scar formation, and systemic metabolic homeostasis require further investigation [70]. Our current *in-vivo* validation of the GAS6/MER/AKT signaling axis relies on pharmacological MER inhibition, which may suffer from potential off-target effects. Future studies will utilize GAS6-specific neutralizing antibodies or genetic knockout models to further confirm the *in-vivo* causal relationship. Finally, beyond small-molecule-loaded therapeutic scaffolds, plant-derived bioactive constituents represent another promising class of diabetic wound-repair agents, with multiple engineered dressing platforms delivering herbal extracts to exert anti-inflammatory and pro-angiogenic functions. Nonetheless, comparable translational bottlenecks-cargo stability, tunable release, and standardized manufacturing-impede clinical translation of both natural-product-based and metabolism-regulating wound dressings [71].

## 5 Conclusion

Diabetic wound healing remains a major clinical challenge due to impaired angiogenesis and a dysregulated immune microenvironment. In this study, AICAR-mediated AMPK activation in ECs enhanced angiogenesis by increasing cellular energy supply. Moreover, AICAR-induced exosomes derived from high-energy-state ECs (AI-EXO) also delivered the paracrine protein GAS6 and reprogrammed macrophages through the GAS6/MER/AKT signaling pathway. Using electrostatic direct-writing technology, we fabricated an AICAR-loaded electrospun scaffold (MEWs + GelMA@AI). After transplantation onto chronic diabetic wounds, the scaffold significantly promoted angiogenesis, improved the local immune microenvironment, induced M1-to-M2 macrophage reprogramming, and ultimately accelerated wound healing. These findings identify a metabolic-energy-mediated, dual-targeted strategy that simultaneously addresses impaired angiogenesis and immune microenvironment dysregulation, providing a new therapeutic perspective for chronic diabetic wounds.

## CRediT authorship contribution statement

**Hengxian Su:** Writing – review & editing, Writing – original draft, Methodology, Investigation, Data curation, Conceptualization. **Chang Liu:** Investigation, Formal analysis, Data curation. **Yusheng Cheng:** Methodology, Conceptualization. **Wenping Cheng:** Formal analysis, Data curation. **Anqi Xu:** Formal analysis, Data curation. **Daoyuan Hu:** Software, Formal analysis, Data curation. **Hongyu Shi:** Software, Formal analysis. **Mengshi Huang:** Software, Formal analysis. **Hao Yuan:** Software, Formal analysis. **Chi Huang:** Software, Formal analysis. **Yaxian Huang:** Formal analysis, Data curation. **Shixing Su:** Investigation, Funding acquisition. **Chuanzhi Duan:** Methodology, Investigation, Funding acquisition. **Xin Feng:** Funding acquisition, Conceptualization.

## Ethics approval and consent to participate

All procedures were approved by the Southern Medical University Ethics Committee (Guangzhou, China; approval no. LAEC-2023-044) and were performed in accordance with the National Institutes of Health guidelines for the care and use of laboratory animals.

## Consent for publication

All authors consented to publication of the manuscript.

## Funding

This work was supported by the 10.13039/501100001809National Natural Science Foundation of China (no. 82571457, 82201427, 82571691, and 82401888), the Foundation of 10.13039/501100018684National Health Commission Capacity Building and Continuing Education Center (no. GWJJ2022100102, and GWJJQ2023100101), the General Program of 10.13039/501100003453Guangdong Provincial Natural Science Foundation (Grant No.2025A1515012753), and the Dongguan Science and Technology of Social Development Program (20221800906152).

## Declaration of competing interest

All authors declare that we do not have any commercial or associative interest that represents a conflict of interest in connection with the work submitted.

## Data Availability

All data generated or analyzed during this study are included in this published article (and its supplementary information files).

## References

[1] Cole J.B., Florez J.C. (2020). Genetics of diabetes mellitus and diabetes complications. Nat. Rev. Nephrol..

[2] Cho N.H., Shaw J.E., Karuranga S., Huang Y., da Rocha Fernandes J.D., Ohlrogge A.W., Malanda B. (2018). IDF diabetes atlas: global estimates of diabetes prevalence for 2017 and projections for 2045. Diabetes Res. Clin. Pract..

[3] Boulton A.J., Vileikyte L., Ragnarson-Tennvall G., Apelqvist J. (2005). The global burden of diabetic foot disease. Lancet.

[4] Xiang P., Jiang M., Chen X., Chen L., Cheng Y., Luo X., Zhou H., Zheng Y. (2024). Targeting grancalcin accelerates wound healing by improving angiogenesis in diabetes. Adv. Sci. (Weinh.).

[5] Fang J., Wang Y., Ling P., Yu D., Hu J., Wang J., Xia Q., Zhu W., Xu W., Shi J., Wu X., Li Y., Zhu X., Fu H., Shou Q., Zhang W., Li S. (2025). Microenvironment self-adaptive multifunctional hydrogels elicit immunomodulation mediated wound healing. Mater. Today Bio.

[6] Gao Q., Hu F., Chai Z., Zheng C., Zhang W., Pu K., Yang Z., Zhang Y., Ramrkrishna S., Wu X., Lu T. (2025). Multifunctional hydrogel with mild photothermal properties enhances diabetic wound repair by targeting MRSA energy metabolism. J. Nanobiotechnol..

[7] Chen W., Xiao W., Liu X., Yuan P., Zhang S., Wang Y., Wu W. (2022). Pharmacological manipulation of macrophage autophagy effectively rejuvenates the regenerative potential of biodegrading vascular graft in aging body. Bioact. Mater..

[8] Xu H., Zhu Y., Hsiao A.W., Xu J., Tong W., Chang L., Zhang X., Chen Y.F., Li J., Chen W., Zhang Y., Chan H.F., Lee C.W. (2023). Bioactive glass-elicited stem cell-derived extracellular vesicles regulate M2 macrophage polarization and angiogenesis to improve tendon regeneration and functional recovery. Biomaterials.

[9] Hu Q., Lyon C.J., Fletcher J.K., Tang W., Wan M., Hu T.Y. (2021). Extracellular vesicle activities regulating macrophage- and tissue-mediated injury and repair responses. Acta Pharm. Sin. B.

[10] Yan L., Wang J., Cai X., Liou Y.C., Shen H.M., Hao J., Huang C., Luo G., He W. (2024). Macrophage plasticity: signaling pathways, tissue repair, and regeneration. MedComm.

[11] Kang Y., Xu L., Dong J., Yuan X., Ye J., Fan Y., Liu B., Xie J., Ji X. (2024). Programmed microalgae-gel promotes chronic wound healing in diabetes. Nat. Commun..

[12] Shu F., Huang H., Xiao S., Xia Z., Zheng Y. (2024). Netrin-1 co-cross-linked hydrogel accelerates diabetic wound healing in situ by modulating macrophage heterogeneity and promoting angiogenesis. Bioact. Mater..

[13] Wang H., Zhou M., Ruan Y., Shen Y., Qin Z., Wu X., Ling H., Ye W., Wang Y., Gan X. (2025). 2A-biohydrogels accelerate diabetic wound healing by promoting M2 macrophage polarization and functionalized mitochondrial transfer to endothelial cells. Chem. Eng. J..

[14] Cai J., Quan Y., Zhu S., Jiayan L., Zhang Q., Liu J., Liang Z., Liao Y., Jiang W., He Y., Su T., Lu F. (2024). The browning and mobilization of subcutaneous white adipose tissue supports efficient skin repair. Cell Metab..

[15] Atcha H., Jairaman A., Holt J.R., Meli V.S., Nagalla R.R., Veerasubramanian P.K., Brumm K.T., Lim H.E., Othy S., Cahalan M.D., Pathak M.M., Liu W.F. (2021). Mechanically activated ion channel Piezo1 modulates macrophage polarization and stiffness sensing. Nat. Commun..

[16] Francos-Quijorna I., Sanchez-Petidier M., Burnside E.R., Badea S.R., Torres-Espin A., Marshall L., de Winter F., Verhaagen J., Moreno-Manzano V., Bradbury E.J. (2022). Chondroitin sulfate proteoglycans prevent immune cell phenotypic conversion and inflammation resolution via TLR4 in rodent models of spinal cord injury. Nat. Commun..

[17] Li Y., Feng J., Song S., Li H., Yang H., Zhou B., Li Y., Yue Z., Lian H., Liu L., Hu S., Nie Y. (2020). gp130 controls cardiomyocyte proliferation and heart regeneration. Circulation.

[18] Su H., Liu W., Li X., Li G., Guo S., Liu C., Yang T., Ou C., Liu J., Li Y., Wei C., Huang Q., Xu T., Duan C. (2023). Cellular energy supply for promoting vascular remodeling of small-diameter vascular grafts: a preliminary study of a new strategy for vascular graft development. Biomater. Sci..

[19] Liu H., Du Y., St-Pierre J.P., Bergholt M.S., Autefage H., Wang J., Cai M., Yang G., Stevens M.M., Zhang S. (2020). Bioenergetic-active materials enhance tissue regeneration by modulating cellular metabolic state. Sci. Adv..

[20] Li Z., Yang B., Yang Z., Xie X., Guo Z., Zhao J., Wang R., Fu H., Zhao P., Zhao X., Chen G., Li G., Wei F., Bian L. (2024). Supramolecular hydrogel with ultra-rapid cell-mediated network adaptation for enhancing cellular metabolic energetics and tissue regeneration. Adv. Mater..

[21] Liu X., Jiang S., Jiang T., Lan Z., Zhang X., Zhong Z., Wu X., Xu C., Du Y., Zhang S. (2024). Bioenergetic-active exosomes for cartilage regeneration and homeostasis maintenance. Sci. Adv..

[22] Zhang X., Lan Z., Jiang S., Liu X., Jiang T., Zhong Z., Li J., Chen C., Wu X., Xu J., Shi Y., Du Y., Zhang S. (2025). A highly versatile bioenergetic-active scaffold system for customized orthopedic applications. Mater. Today.

[23] Li X., Carmeliet P. (2018). Targeting angiogenic metabolism in disease. Science.

[24] Ma H., Jia H., Zou W., Ji F., Wang W., Zhao J., Yuan C., Jiao J. (2024). Gasdermin D mediated mitochondrial metabolism orchestrate neurogenesis through LDHA during embryonic development. Adv. Sci. (Weinh.).

[25] Kosaisawe N., Sparta B., Pargett M., Teragawa C.K., Albeck J.G. (2021). Transient phases of OXPHOS inhibitor resistance reveal underlying metabolic heterogeneity in single cells. Cell Metab..

[26] Radler J., Gupta D., Zickler A., Andaloussi S.E. (2023). Exploiting the biogenesis of extracellular vesicles for bioengineering and therapeutic cargo loading. Mol. Ther..

[27] Xiong M., Zhang Q., Hu W., Zhao C., Lv W., Yi Y., Wang Y., Tang H., Wu M., Wu Y. (2021). The novel mechanisms and applications of exosomes in dermatology and cutaneous medical aesthetics. Pharmacol. Res..

[28] Song Y., You Y., Xu X., Lu J., Huang X., Zhang J., Zhu L., Hu J., Wu X., Xu X., Tan W., Du Y. (2023). Adipose-derived mesenchymal stem cell-derived exosomes biopotentiated extracellular matrix hydrogels accelerate diabetic wound healing and skin regeneration. Adv. Sci. (Weinh.).

[29] Tu Y., Zheng W., Ding Z., Xiang J., Yang Q., Liu Y., Cao J., Shen Y., Tang Z., Lin S., Fan L., Xu Y., Chen B. (2025). Exosome-loaded tannic acid-thioctic acid hydrogel enhances wound healing in coagulation disorders. Mater. Today Bio.

[30] Cheng P., Xie X., Hu L., Zhou W., Mi B., Xiong Y., Xue H., Zhang K., Zhang Y., Hu Y., Chen L., Zha K., Lv B., Lin Z., Lin C., Dai G., Hu Y., Yu T., Hu H., Liu G., Zhang Y. (2024). Hypoxia endothelial cells-derived exosomes facilitate diabetic wound healing through improving endothelial cell function and promoting M2 macrophages polarization. Bioact. Mater..

[31] Pegtel D.M., Gould S.J. (2019). Exosomes. Annu. Rev. Biochem..

[32] Kim J., Zhu Y., Chen S., Wang D., Zhang S., Xia J., Li S., Qiu Q., Lee H., Wang J. (2023). Anti-glioma effect of ginseng-derived exosomes-like nanoparticles by active blood-brain-barrier penetration and tumor microenvironment modulation. J. Nanobiotechnol..

[33] Lu Y., Li H., Wang J., Yao M., Peng Y., Liu T., Li Z., Luo G., Deng J. (2021). Engineering bacteria-activated multifunctionalized hydrogel for promoting diabetic wound healing. Adv. Funct. Mater..

[34] Borcherding N., Brestoff J.R. (2023). The power and potential of mitochondria transfer. Nature.

[35] Liu D., Gao Y., Liu J., Huang Y., Yin J., Feng Y., Shi L., Meloni B.P., Zhang C., Zheng M., Gao J. (2021). Intercellular mitochondrial transfer as a means of tissue revitalization. Signal Transduct. Targeted Ther..

[36] Hardie D.G., Ross F.A., Hawley S.A. (2012). AMPK: a nutrient and energy sensor that maintains energy homeostasis. Nat. Rev. Mol. Cell Biol..

[37] Herzig S., Shaw R.J. (2018). AMPK: guardian of metabolism and mitochondrial homeostasis. Nat. Rev. Mol. Cell Biol..

[38] Wang D., Jiang J., Wang M., Li K., Liang H., Wang N., Liu W., Wang M., Zhou S., Zhang M., Xiao Y., Shen X., Li Z., Wu W., Lin X., Xiang X., Xie Q., Liu W., Zhou X., Tang Q., Zhou W., Yang L., Chuong C.M., Lei M. (2024). Mitophagy promotes hair regeneration by activating glutathione metabolism. Research.

[39] Suomalainen A., Nunnari J. (2024). Mitochondria at the crossroads of health and disease. Cell.

[40] Willenborg S., Sanin D.E., Jais A., Ding X., Ulas T., Nuchel J., Popovic M., MacVicar T., Langer T., Schultze J.L., Gerbaulet A., Roers A., Pearce E.J., Bruning J.C., Trifunovic A., Eming S.A. (2021). Mitochondrial metabolism coordinates stage-specific repair processes in macrophages during wound healing. Cell Metab..

[41] Yu Q., Zheng Z., Zhang H., Xie E., Chen L., Jiang Z., Gao Y., Ma J., Yang L. (2025). Effects of reactive oxygen species and antioxidant strategies on wound healing in diabetes. Interdiscip. Med..

[42] Pena O.A., Martin P. (2024). Cellular and molecular mechanisms of skin wound healing. Nat. Rev. Mol. Cell Biol..

[43] Fu Y.J., Shi Y.F., Wang L.Y., Zhao Y.F., Wang R.K., Li K., Zhang S.T., Zha X.J., Wang W., Zhao X., Yang W. (2023). All-natural immunomodulatory bioadhesive hydrogel promotes angiogenesis and diabetic wound healing by regulating macrophage heterogeneity. Adv. Sci. (Weinh.).

[44] Wang J., Li X., Zhao X., Yuan S., Dou H., Cheng T., Huang T., Lv Z., Tu Y., Shi Y., Ding X. (2024). Lactobacillus rhamnosus GG-derived extracellular vesicles promote wound healing via miR-21-5p-mediated re-epithelization and angiogenesis. J. Nanobiotechnol..

[45] Jin S., Wang Y., Wu X., Li Z., Zhu L., Niu Y., Zhou Y., Liu Y. (2023). Young exosome bio-nanoparticles restore aging-impaired tendon stem/progenitor cell function and reparative capacity. Adv. Mater..

[46] Li C., Ni Y.Q., Xu H., Xiang Q.Y., Zhao Y., Zhan J.K., He J.Y., Li S., Liu Y.S. (2021). Roles and mechanisms of exosomal non-coding RNAs in human health and diseases. Signal Transduct. Targeted Ther..

[47] Zhuang Y., Jiang S., Deng X., Lao A., Hua X., Xie Y., Jiang L., Wang X., Lin K. (2024). Energy metabolism as therapeutic target for aged wound repair by engineered extracellular vesicle. Sci. Adv..

[48] Yu H., Feng H., Zeng H., Wu Y., Zhang Q., Yu J., Hou K., Wu M. (2024). Exosomes: the emerging mechanisms and potential clinical applications in dermatology. Int. J. Biol. Sci..

[49] Jung H., Lee S.Y., Lim S., Choi H.R., Choi Y., Kim M., Kim S., Lee Y., Han K.H., Chung W.S., Kim C.H. (2022). Anti-inflammatory clearance of amyloid-beta by a chimeric Gas6 fusion protein. Nat. Med..

[50] Lu C., Song Y., Wu X., Lei W., Chen J., Zhang X., Liu Q., Deng C., Liang Z., Chen Y., Ren J., Yang Y. (2025). Pleiotropic role of GAS6 in cardioprotection against ischemia-reperfusion injury. J. Adv. Res..

[51] Vullings J., Vago J.P., Waterborg C.E.J., Thurlings R.M., Koenders M.I., van Lent P., van der Kraan P.M., Amaral F.A., van de Loo F.A.J. (2020). Selective increment of synovial soluble TYRO3 correlates with disease severity and joint inflammation in patients with rheumatoid arthritis. J. Immunol. Res..

[52] Nicolas-Avila J.A., Lechuga-Vieco A.V., Esteban-Martinez L., Sanchez-Diaz M., Diaz-Garcia E., Santiago D.J., Rubio-Ponce A., Li J.L., Balachander A., Quintana J.A., Martinez-de-Mena R., Castejon-Vega B., Pun-Garcia A., Traves P.G., Bonzon-Kulichenko E., Garcia-Marques F., Cusso L., N A.G., Gonzalez-Guerra A., Roche-Molina M., Martin-Salamanca S., Crainiciuc G., Guzman G., Larrazabal J., Herrero-Galan E., Alegre-Cebollada J., Lemke G., Rothlin C.V., Jimenez-Borreguero L.J., Reyes G., Castrillo A., Desco M., Munoz-Canoves P., Ibanez B., Torres M., Ng L.G., Priori S.G., Bueno H., Vazquez J., Cordero M.D., Bernal J.A., Enriquez J.A., Hidalgo A. (2020). A network of macrophages supports mitochondrial homeostasis in the heart. Cell.

[53] Schilperoort M., Ngai D., Katerelos M., Power D.A., Tabas I. (2023). PFKFB2-mediated glycolysis promotes lactate-driven continual efferocytosis by macrophages. Nat. Metab..

[54] Vago J.P., Amaral F.A., van de Loo F.A.J. (2021). Resolving inflammation by TAM receptor activation. Pharmacol. Ther..

[55] Zhong F., Chen H., Xie Y., Azeloglu E.U., Wei C., Zhang W., Li Z., Chuang P.Y., Jim B., Li H., Elmastour F., Riyad J.M., Weber T., Chen H., Wang Y., Zhang A., Jia W., Lee K., He J.C. (2018). Protein S protects against podocyte injury in diabetic nephropathy. J. Am. Soc. Nephrol..

[56] Audu C.O., Melvin W.J., Joshi A.D., Wolf S.J., Moon J.Y., Davis F.M., Barrett E.C., Mangum K.D., Deng H., Xing X., Wasikowski R., Tsoi L.C., Sharma S.B., Bauer T.M., Shadiow J., Corriere M.A., Obi A.T., Kunkel S.L., Levi B., Moore B.B., Gudjonsson J.E., Smith A.M., Gallagher K.A. (2022). Macrophage-specific inhibition of the histone demethylase JMJD3 decreases STING and pathologic inflammation in diabetic wound repair. Cell. Mol. Immunol..

[57] Lv D., Cao X., Zhong L., Dong Y., Xu Z., Rong Y., Xu H., Wang Z., Yang H., Yin R., Chen M., Ke C., Hu Z., Deng W., Tang B. (2023). Targeting phenylpyruvate restrains excessive NLRP3 inflammasome activation and pathological inflammation in diabetic wound healing. Cell Rep. Med..

[58] Yang L., Zhang D., Li W., Lin H., Ding C., Liu Q., Wang L., Li Z., Mei L., Chen H., Zhao Y., Zeng X. (2023). Biofilm microenvironment triggered self-enhancing photodynamic immunomodulatory microneedle for diabetic wound therapy. Nat. Commun..

[59] Zhang J., Li W., Liu Y., Zheng J., Liu G., He M., Zheng Z., Zhu M., Cho N., Liang G., Han X., Ying H., Shi Q. (2026). OTUD1 delays wound healing by regulating endothelial function and angiogenesis in diabetic mice. J. Adv. Res..

[60] Deng Q.S., Gao Y., Rui B.Y., Li X.R., Liu P.L., Han Z.Y., Wei Z.Y., Zhang C.R., Wang F., Dawes H., Zhu T.H., Tao S.C., Guo S.C. (2023). Double-network hydrogel enhanced by SS31-loaded mesoporous polydopamine nanoparticles: symphonic collaboration of near-infrared photothermal antibacterial effect and mitochondrial maintenance for full-thickness wound healing in diabetes mellitus. Bioact. Mater..

[61] Sharifiaghdam M., Shaabani E., Faridi-Majidi R., De Smedt S.C., Braeckmans K., Fraire J.C. (2022). Macrophages as a therapeutic target to promote diabetic wound healing. Mol. Ther..

[62] Sun D., Chang Q., Lu F. (2024). Immunomodulation in diabetic wounds healing: the intersection of macrophage reprogramming and immunotherapeutic hydrogels. J. Tissue Eng..

[63] Liu X., Wei Q., Sun Z., Cui S., Wan X., Chu Z., Zhang Y., Zhong W., Lu L., Shi L., Fu X., Zhang C., Wang S. (2023). Small extracellular vesicles: yields, functionalization and applications in diabetic wound management. Interdiscip. Med..

[64] M.A.-O. Tkach, J.A.-O. Kowal, C.A.-O. Théry, Why the need and how to approach the functional diversity of extracellular vesicles, Philos. Trans. R. Soc. Lond. B Biol. Sci. (1471-2970 (Electronic)). 10.1098/rstb.2016.0479 PMC5717434 29158309

[65] Welsh J.A., Goberdhan D.C.I., O'Driscoll L., Buzas E.I., Blenkiron C., Bussolati B., Cai H., Di Vizio D., Driedonks T.A.P., Erdbrugger U., Falcon-Perez J.M., Fu Q.L., Hill A.F., Lenassi M., Lim S.K., Mahoney M.G., Mohanty S., Moller A., Nieuwland R., Ochiya T., Sahoo S., Torrecilhas A.C., Zheng L., Zijlstra A., Abuelreich S., Bagabas R., Bergese P., Bridges E.M., Brucale M., Burger D., Carney R.P., Cocucci E., Crescitelli R., Hanser E., Harris A.L., Haughey N.J., Hendrix A., Ivanov A.R., Jovanovic-Talisman T., Kruh-Garcia N.A., Ku'ulei-Lyn Faustino V., Kyburz D., Lasser C., Lennon K.M., Lotvall J., Maddox A.L., Martens-Uzunova E.S., Mizenko R.R., Newman L.A., Ridolfi A., Rohde E., Rojalin T., Rowland A., Saftics A., Sandau U.S., Saugstad J.A., Shekari F., Swift S., Ter-Ovanesyan D., Tosar J.P., Useckaite Z., Valle F., Varga Z., van der Pol E., van Herwijnen M.J.C., Wauben M.H.M., Wehman A.M., Williams S., Zendrini A., Zimmerman A.J., Consortium M., Thery C., Witwer K.W. (2024). Minimal information for studies of extracellular vesicles (MISEV2023): from basic to advanced approaches. J. Extracell. Vesicles.

[66] Liu X., Chen K., Zhao X., Zhuang Z., Ni X. (2026). Current research status and progress of laser therapy in wound healing. PLAST AESTHET RES.

[67] Sheckley M., Lowney M., Hahn W.W., Chen K., Gurtner G.C. (2026). Role of mechanical forces and skin tension in scar pathophysiology. PLAST AESTHET RES.

[68] Barakat M., DiPietro L.A., Chen L. (2021). Limited treatment options for diabetic wounds: barriers to clinical translation despite therapeutic success in murine models. Adv. Wound Care.

[69] Ding T., Du Y., Yang B., Tian W., Li J., Xie J. (2025). Comprehensive review of macrophage models: primary cells and immortalized lines across species. Front. Immunol..

[70] Dickenson M.E., Oakes R.S., Morris A.H. (2026). Don't judge an implant by its cover: how the foreign body response and fibrotic capsule might be harnessed for good. NPJ Biomed Innov.

[71] Yang B., Xu J., Liu S., Wu C., Li Y., Lv M., Chen T., Zhao C., Pu D., Tang C., Malashicheva A., Zang G., Wang G. (2025). Applications of bioactive herbal extracts in dressing materials for skin wound repair: ingredients, mechanisms and innovations. Interdiscip. Med..

