# Supplementary material for: AICAR-induced energy supply and paracrine activity of endothelial cells accelerate chronic diabetic wound healing by promoting angiogenesis and modulating macrophage reprogramming
Source: Mater Today Bio. 2026 Oct 9;41:103743. doi: 10.1016/j.mtbio.2026.103743 (PMC13679375; doi:10.1016/j.mtbio.2026.103743)
Supplement: Supplementary file 1 [file mmc1.docx]

**Title:** **AICAR-induced energy supply and paracrine activity of endothelial cells accelerate chronic diabetic wound healing by promoting angiogenesis and modulating macrophage reprogramming**

Hengxian Su^#, 1^, Chang Liu^#, 2^, Yusheng Cheng^#, 3^, Wenping Cheng^1^, Anqi Xu^1^, Daoyuan Hu^4^, Hongyu Shi^1^, Mengshi Huang^1^, Hao Yuan^1^, Chi Huang^1^, Yaxian Huang^1^, Shixing Su^*, 1^, Chuanzhi Duan^*, 1^ and Xin Feng^*, 1^

1. Neurosurgery Center, Department of Cerebrovascular Surgery, National Key Clinical Specialty, Engineering Research Center for Diagnostic and Therapeutic Technology and Devices for Cerebrovascular Diseases under the Ministry of Education, Guangdong Provincial Key Laboratory for Brain Function Repair and Regeneration, Zhujiang Hospital Institute for Brain Science and Intelligence, Zhujiang Hospital, Southern Medical University, Guangzhou 510282, China.
2. Department of Orthopedic Surgery, The Lingnan Hospital of Sun Yat-Sen University, Guangzhou, 510630, China.
3. Department of General Surgery, The Second Clinical Medical College, Lanzhou University, Lanzhou, 730030, China
4. Department of Urology, Biomedical Innovation Center, The Sixth Affiliated Hospital, Sun Yat-Sen University, Guangzhou, 510655, China.

^#^Equal contribution by authors.

*Corresponding authors: Xin Feng, [13681134001@163.com](mailto:13681134001@163.com); Chuanzhi Duan, [doctor_duanzj@163.com](mailto:doctor_duanzj@163.com); Shixing Su, [sushixing001@163.com](mailto:sushixing001@163.com).

1. **Materials and Methods**
1.1. **Glucose concentration detection in chronic diabetic wound tissues and tail vein blood**

For glucose concentration detection in diabetic wound tissues, approximately 20 mg of frozen wound tissue was homogenized in ice-cold PBS. Homogenates were centrifuged at 12000 × g for 10 min at 4 °C, and supernatants were collected for analysis. Glucose concentrations were quantified using a colorimetric glucose assay kit based on the GOD/POD method (Beyotime, Shanghai, China) following the manufacturer’s instructions. Absorbance was read at 505 nm on a microplate reader, and sample glucose levels were calculated against a standard curve. Data were normalized to tissue wet weight and expressed as μmol per gram of tissue.

Fasting blood glucose levels in the rats’ tail vein blood were determined using a colorimetric glucose assay kit based on the GOD/POD method (Beyotime, Shanghai, China). Briefly, peripheral blood samples were collected from the rat tail vein after 6–8 h of fasting. The obtained blood samples were centrifuged to collect serum, and subsequent glucose detection was performed strictly in accordance with the manufacturer’s instructions.

1.2. **Detection of intra-exosomal AICAR via LC-MS/MS**

Purified AI-EXO samples were fully lysed, and 2 mL of the lysate was filtered through a 0.22 μm membrane to prepare the test solution. A reference standard stock solution was prepared by dissolving accurately weighed AICAR powder in methanol to a final concentration of 1 μg/mL (1 ppm).

All measurements were performed on a Waters XEVO TQ-S Crons 2 liquid chromatography-tandem mass spectrometry (LC-MS/MS) system controlled by MassLynx 4.2 software. For chromatographic separation, the mobile phase consisted of (A) aqueous solution containing 0.01% formic acid and 25 mmol/L ammonium formate and (B) acetonitrile. Gradient elution was carried out at a flow rate of 0.3 mL/min, with the column temperature maintained at 40 °C and an injection volume of 10 μL.

Mass spectrometric detection was conducted in positive electrospray ionization (ESI+) mode. The capillary voltage was set at 4000 V, and the ion source temperature was kept at 350 °C. Targeted quantitative and qualitative detection was achieved in multiple reaction monitoring (MRM) mode with the following transitions: m/z 259.2 → 109.9 (collision energy: 40 V for quantification) and m/z 259.2 → 126.9 (collision energy: 10 V for confirmation).

The presence of AICAR in exosome preparations was determined by comparing retention time and MRM signal intensity with the reference standard. A sample with no detectable target peak above baseline noise was defined as AICAR-negative

1.3. **Quantitative analysis of Encapsulation Efficiency (EE) and drug loading content (DLC) of AICAR in MEWs+GelMA@AI scaffolds**

The Encapsulation Efficiency (EE) and drug loading content (DLC) of AICAR in MEWs+GelMA@AI scaffolds were quantitatively determined via an established method[1]. In brief, AICAR was dissolved in PBS to prepare standard dilutions ranging from 0.0195 mM to 5 mM. The absorbance values of standard dilutions were determined with a NanoDrop spectrophotometer (Waltham, MA, USA) and applied to create a standard curve. Based on the standard curve (R^2^ > 0.999), the AICAR concentration of each sample was determined.

For sample preparation, dried MEWs+GelMA@AI scaffolds (n = 6) were accurately weighed and immersed in 2 mL of PBS. The mixtures were incubated in a thermostatic shaker at 37 °C at 150 rpm for 24 h to fully extract the loaded AICAR from the GelMA hydrogel network. After incubation, the supernatants were collected by centrifugation at 8000 rpm for 10 min. The absorbance of each supernatant was measured at 260 nm.

The total mass of the loaded AICAR was calculated using the standard curve. Encapsulation Efficiency (EE) and drug loading content (DLC) were calculated using the following formulas:

Encapsulation Efficiency (%) =m _loaded_/m _initial_ × 100%

Drug Loading Content (μg/mg) =m _loaded_/m _solid_

where m _loaded_ is the total mass of AICAR loaded in the scaffold, m _initial_ represents the total mass of AICAR initially added during scaffold fabrication, and m _solid_ is the dry weight of the composite scaffold and GelMA.

Fig. S1. Effect of gradient particle concentrations of AI-EXO on RAW264.7 cell viability. RAW264.7 cells were seeded into 96-well plates at 5×10³ cells/well and cultured at 37 °C with 5% CO₂ for 12 h. Cells were treated with a gradient of AI-EXO concentrations (1 × 10⁷ to 5 × 10⁹ particles/mL) or an equal volume of sterile PBS for 24 hours. Cell viability was determined using a CCK-8 kit by measuring absorbance at 450 nm. All results are shown as mean ± SD. n = 6. Experiments were repeated 3 times independently; ns = no statistical significance. One-way ANOVA followed by Tukey’s post hoc test was used for comparisons among multiple groups. **P < 0.01, ***P < 0.001.

Fig. S2. Gating strategy for flow cytometry analysis of macrophage polarization. Cellular debris was first excluded based on FSC-H and SSC-H signals. Single-cell populations were further gated using FSC-A versus FSC-H to exclude cell aggregates. F4/80-positive macrophages were identified among single cells, and the proportions of iNOS⁺ (M1) and Arg-1⁺ (M2) subsets were quantified.

Table S1. Primers used for RT-qPCR

| Gene | Primer Sequence (5′ → 3′) | PCR Product (bp) | |
| --- | --- | --- | --- |
| iNOS | F: CAGCTGGGCTGTACAAACCTT | | 195 |
|  | R: CATTGGAAGTGAAGCGTTTCG | |  |
| Arg-1 | F: CTCCAAGCCAAAGTCCTTAGAG | | 150 |
|  | R: AGGAGCTGTCATTAGGGACATC | |  |
| GAPDH | F: GCACAGTCAAGGCCGAGAAT | | 151 |
|  | R: GCCTTCTCCATGGTGGTGAA | |  |

F, forward; R, reverse;

Fig. S3. A) Rat tail vein blood glucose was monitored for 2 weeks post-STZ injection. Animals with sustained blood glucose levels > 16.7 mM were confirmed to be valid chronic diabetic models. B) The measurement of local glucose concentrations in wound tissues across all experimental groups at days 3, 7, and 14 post-wounding, with an additional normal non-diabetic control group included for baseline reference. n = 6. All results are shown as mean ± SD. Experiments were repeated 3 times independently. One-way ANOVA followed by Tukey’s post hoc test was used for comparisons among multiple groups. ***P < 0.001.

Fig. S4. A, B) Quantitative analysis of JC-1 immunofluorescence staining (A) and MitoSOX Red staining (B). All results are shown as mean ± SD. n = 5, Experiments were repeated 3 times independently, ns = no statistical significance. One-way ANOVA followed by Tukey’s post hoc test was used for comparisons among multiple groups. **P < 0.01, ***P < 0.001.

Fig. S5. Targeted LC-MS/MS analysis revealed no quantifiable AICAR in purified AI-EXO lysates. A) Reference standard; B) AI-EXO lysate supernatant.

Fig. S6. A, B) Particle yield and protein amount comparison between AI-EXO and N-EXO (n = 6). Experiments were repeated 3 times independently. All results are shown as mean ± SD. An unpaired two-tailed Student’s t-test was used for comparisons between the two groups. ***P < 0.001.

Fig. S7. A, B) Proteomic analysis of Normal- and AICAR-EXO (N-EXO and AI-EXO). A) Kyoto Encyclopedia of Genes and Genomes (KEGG) analysis revealed numerous proteins with significant differential expression between N- and AI-EXO that are related to energy metabolism signaling pathways, including fatty acid metabolism (blue arrow), oxidative phosphorylation (blue arrow), citrate cycle, etc. B) Gene Ontology (GO) analysis demonstrated that the significantly enriched GO terms included ATP-dependent protein folding chaperone (MF), collagen-containing extracellular matrix (CC), and wound healing (BP).

Fig. S8. A) Si-GAS6 #3 was the most effective GAS6 knockdown among the three siRNAs for HUVEC, as assessed by WB. B) Quantitative analysis of the relative GAS6 protein expression. All results are shown as mean ± SD. Experiments were repeated 3 times independently, ns = no statistical significance. One-way ANOVA followed by Tukey’s post hoc test was used for comparisons among multiple groups. *P < 0.05, **P < 0.01, ***P < 0.001.

Fig. S9. AI-EXO elevated the proportion of M2 phenotype macrophages in IL-4-induced RAW264.7 cells. A) Immunofluorescence staining of Arg-1 (M2 marker) and iNOS (M1 marker). Scale bar = 50 μm. B) Quantitative analysis of Arg-1 and iNOS mRNA expression (n = 5). All results are shown as mean ± SD. Experiments were repeated 3 times independently; ns = no statistical significance. One-way ANOVA followed by Tukey’s post hoc test was used for comparisons among multiple groups. *P < 0.05, **P < 0.01, ***P < 0.001.

Fig. S10. GAS6-specific neutralization (GAS6 nAb) reverses AI-EXO-mediated M2 macrophage reprogramming. A) Representative immunofluorescence images of iNOS (M1 marker, red) and Arg-1 (M2 marker, green) in LPS-primed RAW264.7 macrophages receiving the indicated treatments. Nuclei were counterstained with DAPI (blue). Scale bar = 50 μm; B) Flow cytometry plots showing the percentages of iNOS⁺ M1 and Arg-1⁺ M2 macrophages in each group; C) Western blot analysis of iNOS and Arg-1 protein levels in macrophages from each group. All results are shown as mean ± SD. Experiments were repeated 3 times independently; ns = no statistical significance. One-way ANOVA followed by Tukey’s post hoc test was used for comparisons among multiple groups. *P < 0.05, **P < 0.01, ***P < 0.001.

Fig. S11. Compressive modulus and equilibrium swelling ratio of pure GelMA, MEWs, MEWs + GelMA, and MEWs + GelMA@AI. A) Compressive modulus was assessed via quasi‑static uniaxial compression using a universal mechanical tester. B) The equilibrium swelling ratios were calculated. All results are shown as mean ± SD. n = 6, Experiments were repeated 3 times independently, ns = no statistical significance. One-way ANOVA followed by Tukey’s post hoc test was used for comparisons among multiple groups. **P < 0.01, ***P < 0.001.

Table S2. The data of Encapsulation Efficiency (EE) and drug loading content (DLC)

| sample | 1 | 2 | 3 | 4 | 5 | 6 |
| --- | --- | --- | --- | --- | --- | --- |
| EE (%) | 75.32 | 73.41 | 74.88 | 80.08 | 83.74 | 78.62 |
| DLC (μg/mg) | 1.04 | 0.99 | 1.01 | 1.10 | 1.17 | 1.09 |

Table S2. The Encapsulation Efficiency (EE) and drug loading content (DLC) of AICAR in MEWs+GelMA@AI scaffolds were measured. Quantitative analysis showed that the Encapsulation Efficiency of AICAR and the drug loading content of the MEWs+GelMA@AI scaffolds were 77.68 ± 3.87 % and 1.07 ± 0.07 μg/mg, respectively (mean ± SD, n = 6).

Fig. S12. Systemic metabolic impact of AICAR. Fasting blood glucose (A) and body weight (B) of all rats throughout the experiment. n = 6. All results are shown as mean ± SD. Experiments were repeated 3 times independently, ns = no statistical significance. One-way ANOVA followed by Tukey’s post hoc test was used for comparisons among multiple groups.­­

Fig. S13. The effects of MEWs+GelMA@AI on the biosafety of rats were investigated by HE staining of major organs. H&E staining of major organs showed no significant intergroup histopathological differences or detectable tissue abnormalities, confirming the favorable *in vivo* biosafety of the composite scaffold.

Fig. S14. Diabetic wound metabolism at days 3, 7, and 14 post-wounding. MEWs+GelMA@AI group showed an elevated NAD⁺/NADH ratio (A) and reduced lactate/pyruvate ratio (B) relative to the other groups, indicating that local AICAR delivery ameliorates wound metabolic dysfunction. n = 3. All results are shown as mean ± SD. Experiments were repeated 3 times independently; ns = no statistical significance. One-way ANOVA followed by Tukey’s post hoc test was used for comparisons among multiple groups. *P < 0.05, **P < 0.01, ***P < 0.001.

**Reference:**

[1] H. Su, W. Liu, X. Li, G. Li, S. Guo, C. Liu, T. Yang, C. Ou, J. Liu, Y. Li, C. Wei, Q. Huang, T. Xu, C. Duan, Cellular energy supply for promoting vascular remodeling of small-diameter vascular grafts: a preliminary study of a new strategy for vascular graft development, Biomater Sci 11(9) (2023) 3197-3213.
